# A systematic review of the pivotal role of environmental toxicant exposure on infectious diseases in low- and middle-income countries

**DOI:** 10.1016/j.puhip.2025.100631

**Published:** 2025-06-25

**Authors:** Rehnuma Haque, Md Shariful Islam, Molly Hanson, Md. Zamiur Rahaman, Sadia Afrin, Sristi Shome, Mahbubur Rahman, Syed Moshfiqur Rahman, KM Saif-Ur-Rahman, Rubhana Raqib

**Affiliations:** a Environmental Health and WASH, Health Systems and Population Studies Division, icddr,b, Dhaka, 1212, Bangladesh; b The School of Public Health, The University of Queensland, Brisbane, Qld, Australia; c Global Health and Migration Unit, Department of Women's and Children's Health, Uppsala University, Uppsala, Sweden; d College of Medicine Nursing and Health Sciences, University of Galway, Galway, Ireland; e Evidence Synthesis Ireland and Cochrane Ireland, University of Galway, Galway, Ireland; f Nutrition Research Division, icddr,b, Dhaka, 1212, Bangladesh; g Maternal and Child Health Division, icddr,b, Dhaka, 1212, Bangladesh

## Abstract

**Objective:**

The objective of this review is to identify which environmental toxicants are linked to infectious diseases in low- and middle-income countries (LMICs) by synthesizing available evidence. It aims to summarize key findings, identify research gaps and provide policy recommendations based on the associations between specific toxicants and disease outcomes.

**Study design:**

Systematic review.

**Methods:**

We conducted a comprehensive search in PubMed, Scopus, Web of Science (core collection), and CENTRAL (the Cochrane Library) to identify studies on bacterial, viral, and parasitic pathogenic activity.

**Result:**

This search yielded 11,468 studies, of which 55 met inclusion criteria after screening following the standard methods. A consistent association is found between particulate matter (PM2.5 and PM10) exposure and increased respiratory infection risk, with PM2.5 particularly linked to increased incidence and mortality in diseases like COVID-19 and tuberculosis. Heavy metals, including lead, cadmium, and mercury worsen chronic infections such as HIV/AIDS and hepatitis by increasing body burden and inflammation. The review highlights air pollutants’ substantial impact on infectious disease spread and severity while noting a research gap on other pollutants including persistent organic pollutants (POPs) and polycyclic aromatic hydrocarbons (PAHs). Bias assessment indicates around half the studies show low risk of bias; however, potential biases were noted in confounding variables and blinding of outcome assessment.

**Conclusion:**

The systematic review emphasizes the need for research on biological mechanisms underlying these associations and the impacts of other pollutants. Findings advocate for reducing environmental pollution exposure in LMICs to mitigate infectious disease risk.

## Graphical abstract

**Figure:**
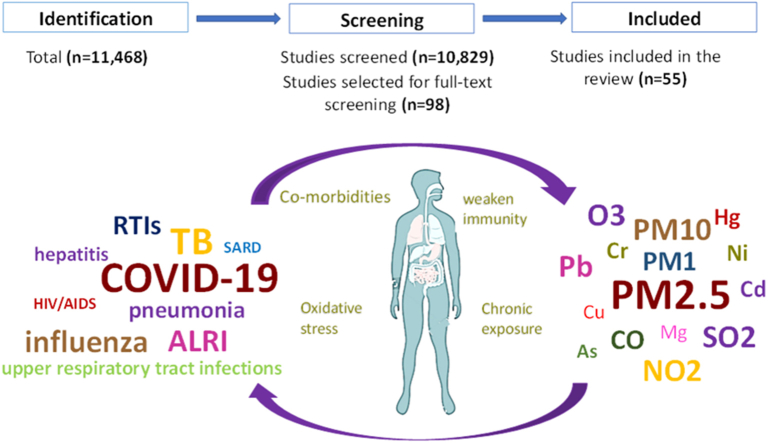

## 1 Introduction

Pollution accounts for one in six deaths worldwide, or around 9 million yearly deaths [1]. The majority of these pollution-related deaths are caused by exposure to common environmental toxicants such as ambient and household air pollutants, water pollutants and hazardous chemical toxicants (e.g., lead) [1]. These environmental pollutants are toxic to the immune system, and persistent exposure contributes to the development of infectious diseases [2]. Low- and middle-income countries (LMICs) share a dual burden of high infectious disease prevalence, including respiratory infections, pneumonia, tuberculosis (TB), HIV/AIDS, neonatal infections, diarrhoea [3] and widespread environmental pollution.

While environmental toxicants such as air pollutants, heavy metals, pesticides, and persistent organic pollutants are known to impair immune function and increase vulnerability to disease, their role in the development, persistence, transmission, and severity of infectious diseases remains underexplored. Current research disproportionately focuses on non-communicable diseases and chemical exposures in high-income settings [4], with limited attention to the environmental determinants of infectious diseases in LMIC contexts. LMICs are severely affected due to weak pollution control policies, unchecked rapid industrialization, and urbanization. Infection risk is also high in areas with a high prevalence of poverty, malnutrition, poor living conditions, and a lack of access to clean water, sanitation, and hygiene (WASH) facilities. Therefore, high infectious disease-related mortality persists in LMICs despite significant advancements in medical research and therapies [2].

Emerging evidence suggests that pollutants such as PM2.5, arsenic (As), lead (Pb), and Cadmium (Cd) can damage epithelial barriers, reduce vaccine efficacy and modulate host immune responses, thereby increasing susceptibility to infections such as tuberculosis, pneumonia, and diarrhoeal diseases. A cohort study in rural Bangladesh by Rahman et al. (2011) provides robust epidemiological evidence linking prenatal arsenic exposure to a heightened risk of infectious diseases during infancy [5]. Similarly, a large population-based study in the United States by Cardenas et al. (2015) [6], using pooled data from NHANES (2003–2004 and 2009–2010), found that higher urinary arsenic concentrations were significantly associated with reduced varicella zoster virus (VZV) IgG seropositivity, indicating impaired immune memory [6]. In addition, Grandjean et al. (2012) [7] reported that elevated exposure to perfluorinated compounds (PFCs) was associated with diminished humoral immune responses to routine childhood vaccines in children aged 5 and 7 years [7]. Despite these important findings, the evidence remains fragmented, and no comprehensive synthesis has been conducted to systematically assess which environmental toxicants are most strongly associated with infectious disease outcomes in LMICs. To address these gaps, this systematic review aims to investigate which environmental toxicants are consistently associated with infectious disease outcomes in LMICs. By synthesizing available evidence, this review will contribute to a better understanding of the environment–infection nexus in LMICs by data extraction on pollutant type and infectious disease outcome, and conclude with a discussion of key implications, research gaps, and policy recommendations.

## 2 Methods

### 2.1 Protocol and registration

The systematic review protocol was registered with PROSPERO (CRD4202127435) and published in 2022 [8]. To conduct this review, we followed the standard guidelines and reported as per the Preferred Reporting Items for Systematic Review and Meta-Analysis (PRISMA) 2020 guidelines (Table S1) [9].

### 2.2 Search strategy

We searched CENTRAL (the Cochrane Library), Scopus, Web of Science (core collection), and PubMed (MEDLINE, PubMed Central (PMC), and Bookshelf) on March 30, 2021 (Table S2). We updated the search on April 25, 2024. The librarian from Uppsala University assisted in reviewing the draft search strategy, ensuring the search is thorough, unbiased, and reproducible through their expertise in information retrieval and management. The inclusion criteria were followed to decide on subject header phrases and keywords included a broad range of environmental and health-related terms to capture relevant literature. Environmental exposure terms encompassed "Toxicants," "Heavy metals," "Pesticides," "Phthalates," "Organochlorine Compounds" or "OCPs," and "Perfluoroalkylated Substances" or "PFAS." Infectious disease terms included "Communicable Diseases," "Blood-borne Infections," "Enteric disease," "Bacterial Diseases," "Parasitic Diseases," "Pneumonia," "Influenza" or "flu," "hepatitis," "Common cold," "Covid 19," "infections", etc. Socioeconomic and geographic terms used to capture LMIC contexts included ","developing country," "poor populations," "poor nation," "middle income economy," "middle income country," "low income populations," "lower income countries," "underserved nations," "emerging countries," "emerging country," and "emerging economy." etc. We also reviewed the citations of the included papers. Any duplicate papers were eliminated using Covidence [10]. The search strategy for different databases has been published previously in the protocol paper [8].

### 2.3 Inclusion criteria

• We included studies that explicitly investigated the relationship between environmental toxicants and infectious diseases, using the PECO (Population, Exposure, Comparator, Outcome) framework to guide the selection process.
• Eligible study designs included cohort studies, cross-sectional studies, case-control studies and randomized controlled trials (RCTs) focused on bacterial, viral, and parasite pathogenic activity. However, in environmental health, RCTs, are rare due to ethical and logistical constraints. Including a wide range of observational and experimental designs allows for comprehensive evidence synthesis and improves the generalizability and robustness of the findings.
• Included studies reported on the influence of environmental toxicants on the emergence, severity, and transmission of infectious diseases. This criterion addresses a critical public health concern by capturing the full spectrum of how environmental pollutants may affect infectious disease dynamics.
• We included studies conducted in LMICs based on (World Bank 2019–20 classification) [11], including both community-based and hospital-based research. This geographic focus targets regions with the highest burden of environmental exposure and infectious diseases, enhancing the relevance of findings for global health policy and intervention strategies.
• No restrictions were applied based on age, sex, gender, ethnicity, religion, health status, or socioeconomic status, to ensure inclusivity, recognizing that environmental toxicants can affect individuals across all demographic groups and life stages.

### 2.4 Exclusion criteria

• We excluded articles such as reviews, systematic reviews, meta-analyses, reviews of reviews, books, chapters, comments, perspectives, editorials, letters to the editor, correspondences, conference proceedings, and opinions as these publications often do not provide original empirical data and were excluded to ensure that the review is based solely on primary research studies, which allow for direct analysis of exposure-outcome relationships.
• Studies where the relationship between environmental toxicants and infectious diseases was not analysed or reported separately were excluded. These studies were excluded because they did not provide distinct data or findings on the connection between toxicant exposure and infectious disease outcomes, limiting their relevance to the research question.
• Studies conducted in high-income countries (HICs) were excluded. These studies were excluded to ensure contextual relevance. The environmental exposure pathways, regulatory environments, healthcare infrastructure, and infectious disease burdens in HICs differ substantially from those in LMICs.

### 2.5 Screening and data extraction

Two independent reviewers screened the title and abstract using Covidence [10] based on the inclusion and exclusion criteria to identify relevant papers for full-text screening. The full-text screening was also performed by the two independent reviewers, and reasons for exclusion were reported based on prioritization and sequential exclusion technique [12]. A third reviewer was consulted in the event of potential conflicts, and the final decision was made. To minimize bias, the two team members independently extracted data in a predetermined format on the study objectives, study population, intervention, and results from the chosen studies. Data extraction generated information on the title, authors, geographic location (countries, cities, and urban/rural areas), publication year, and outcome findings in a structured spreadsheet. After data extraction, both reviewing authors re-examined the data for consistency. Any discrepancies were discussed and assessed by a third reviewer.

### 2.6 Risk of bias assessment

We assessed the risk of bias in the included studies using the risk-of-bias assessment tool for non-randomized studies (RoBANS) [13]. We assessed the risk of bias based on the following domains: (i) selection of participants, (ii) confounding variables, (iii) measurement of exposure, (iv) blinding of outcome assessments, (v) incomplete outcome data, and (vi) selective outcome reporting. We rated the risk of bias as low risk of bias, unclear risk of bias, and high risk of bias.

### 2.7 Data synthesis

A narrative synthesis was conducted following the review objective and outcome measures. We have mapped and summarized the available evidence. Narrative synthesis was performed using three steps. The steps are logical organization of the studies, within-study synthesis, and cross-study synthesis. Firstly, the included studies were logically organized based on key elements such as study design, population, study setting, and main outcomes. Secondly, a within-study synthesis was performed to summarize the main findings, methodologies, and conclusions of each study. Finally, a cross-study synthesis was carried out to identify similarities and differences across the studies, establishing a broader interpretation of the overall evidence. Although we did not conduct a meta-analysis, we considered the heterogeneity of the included studies by comparing similarities and differences in study design, main outcomes (such as prevalence percentages, relative risks (RR), odds ratios (OR), hazard ratios (HR), confidence intervals (CI) and the variety of reported toxicants, as presented in Table 1 and Table S6.

**Table 1:** Characteristics of exposure and outcome in the included studies.

Author, Year, Country	Study Design	Study Period	Exposure	Disease/Infection	Main Outcome
Liang et al. 2014; China [15]	Cross-Sectional	2008–2013	PM2.5	Influenza	In Beijing, from 2008 to 2011, the association between monthly PM2.5 (μg/m^3^) and reported influenza cases showed that PM2.5 leads to influenza by approximately 90°, indicating a 1-2-month delay in the occurrence of influenza relative to PM2.5 levels.
Rivas-Santiago et al., 2015; Mexico [16]	Cross-Sectional	2012	PM2.5, PM10	TB	Data highlights significant differences between unexposed and PM-exposed cells with asterisks indicating a statistical significance (P < 0.05 or P < 0.01).
Memon et al. 2017; Pakistan [17]	Case-Control	2014–2015	Cr, Ni, As, Cd, Pb	TB	TB cases showed increased mean levels of toxic metals compared to controls, with Pb^2+^ at 389 vs 119, Cr^2+^ at 32 vs 12, Cd^2+^ at 5 vs 2.24, and Ar^2+^ at 7.4 vs 1.9.
Bates et al. 2018; Nepal [18]	Case-Control	2006–2007	PM2.5	ALRIs	The adjusted ORs for PM2.5 exposure show an increase in risk, with 1.51 for Q2, 2.22 for Q3, and 2.48 for Q4 in quartile-specific conditional logistic regression. 95 % CI for Q2, Q3 and, Q4 are (1.00, 2.27), (1.47,3.38) and (1.63, 3.77).
Emokpae and Mbonu 2018; Benin [19]	Case-Control	2016	Cr, Ni, Cd, Hg, Pb	AIDS	HIV-positive patients had higher mean levels of toxic metals compared to controls, with Pb^2+^ at 1.22 vs 0.57 μg/dl, Cd^2+^ at 0.62 vs 0.10 μg/dl, and Hg^2+^ at 0.08 compared with 0.04 μg/dl.
S. Zhu et al. 2018; China [20]	Time-series analysis	2010–2015	PM10, NO2, SO2	TB	For each 10 μg/m^3^ increase above the thresholds, the OR increases by 1.06 (6 %) for PM10 (above 70 μg/m^3^) and NO2 (above 40 μg/m^3^), and by 1.07 (7 %) for SO2 (above 60 μg/m^3^). 95 % CI for PM 10, NO2 and, SO2 are (1.01–1.11), (1.03–1.09), and (1.02–1.12).
Zhao et al. 2019; China [21]	Cross-Sectional	2013–2017	CO	Pneumonia	An IQR increase of 0.28 mg/m^3^ in ambient CO concentrations at lag03 was associated with a rise in estimated risk for respiratory hospital outpatient visits by 5.62 %, 8.86 %, 6.67 %, and 7.20 %.
Mokoena et al. 2019; China [22]	Cross-Sectional	2014–2016	PM2.5, SO2, O3	Respiratory diseases	The ORs measured as 1.328 for PM2.5, 2.061 for O3, and 1.524 for SO2. 95 % Cis for PM2.5, O3, and SO2 are (1.033, 1.745), (0.630, 10.198) and (0.836,3.406).
				Pneumonia, Influenza	
Dastoorpoor et al., 2019 ;Iran [23]	Cross-Sectional	2008–2018	PM2.5	Respiratory diseases	For O3, respondents aged 65–74 showed a slight increase in risk at lag 1 with an RR of 1.010 while others aged 75 and > 75 posed a similar risk increase at lag 0 with an RR of 1.004. For PM2.5, men show consistently low-risk changes in multiple lags with an RR of 1.001 while women show a minimal increase at lag 3 with an RR of 1.002.
Aslam et al. 2019; Pakistan [24]	Cross-Sectional	–	Pb, Ni, Mg^2+^,Cu^2+^, Co^2+^, Cd^2+^	Hepatitis C	The significant reduction of the heavy metal levels after Chelation therapy in the blood of the patients, as indicated by p-values <0.05 in all metals.
BS Zhang et al. 2019; China [25]	Cross-Sectional	2004–2016	PM10, NO2, SO2	TB	The report showed a strong positive correlation with PM10 concentration (0.79), a moderate correlation with SO2 concentration (0.64), and a weak correlation with NO2 concentration (0.16).
Davila Cordova et al. 2020; Peru [26]	Cross-Sectional	2011–2015	PM2.5	ALRI, Pneumonia	Rate ratios for respiratory diseases associated with each interquartile range increase in PM2.5 for all Pneumonia, acute lower respiratory infections, and Asthma: <5 year = 1.17, 1.06, and 1.10.
Yuan et al., 2020; China [27]	Cross-Sectional	2020	PM2.5, PM10, SO2, NO2, CO	COVID-19	The correlation resulted in 0.28 of the fixed effect of PM2.5 during lockdown while during the main phase and over the study period, it was 0.20 and 0.19.
Roux et al., 2020; Brazil [28]	Cross-Sectional	–	PM2.5	SARD	With a Spearman's correlation coefficient of 0.52, the study shows that PM2.5 and SARD are positively correlated in all regions of Brazil, except for the south.
Ruchiraset and Tantrakarnapa 2022; Thailand [29]	Longitudinal	2003–2014	PM10, CO, NO2, SO2, O3	Pneumonia	The incidence RRs indicate that a 1 ppb increase in ozone is associated with approximately a 1% decrease in weekly pneumonia cases.
Wu, Zhan, and Zhao 2021; China	Cross-Sectional	2019–2020	PM2.5, PM10, NO2, O3, SO2, CO	COVID-19	1 μg m–3 increase of PM2.5, PM10, and NO2 was correlated to 1.95%, 0.55%, 4.63% increase of COVID- 19, and 2.05% decrease of Covid-19. And 1 μg m–3 increase in O3 is detected as related to 2.05% decrease in COVID-19 morbidity counts.
X. Zhang et al. 2021; China [30]	Cross-Sectional	2020	PM2.5, PM10, NO2, CO, SO2, O3	COVID-19	Significant positive associations were observed between short-term exposure to PM2.5, PM10, and NO2 and the number of regularly new confirmed COVID-19 cases.
Meng et al. 2021; China [31]	Observational	2015–2017	SO2, NO2, O3, PM10, PM2.5	Influenza	If 10 μg/m^3^ increase in concentrations, the RRs at lag 0 are 1.009 for SO2, 1.039 for NO2, 1.005 for O3, 0.998 for PM10, and 0.989 for PM2.5, while at lag 0–1, the RRs are 1.097 for SO2, 1.039 for NO2, 1.005 for O3, 0.996 for PM10, and 0.989 for PM2.5.
Carrasco-Escobar et al. 2020; Peru [32]	Cross-Sectional	2015–2017	PM2.5	TB	Significant associations were identified in the study between poverty level, PM2.5, TB cases, and the three variables combined (Kendall's W = 0.4606; P = 0.001).
Zhu et al., 2021; China [33]	Cross-Sectional	2014–2017	PM1, PM2.5	Respiratory Disease, Pneumonia	Per IQR increase in PM1, the corresponding Excess Risk was 12.21 % of total respiratory, 20.64 % of COPD, and 21.44 % of pneumonia mortality, respectively. Per IQR increase in PM2.5 the corresponding Excess Risk was 12.09 % of total respiratory, 17.70 % of COPD, and 25.74 % of pneumonia mortality, respectively.
P. Zheng et al. 2021; China [34]	Cross-Sectional	2019	NO2, PM2.5, PM10	COVID-19	The increases of 10 μg/m^3^ in concentrations of PM2.5, PM10, and NO2 were found to be significantly correlated with a 32.3 %, 14.2 %, and 37.8 % rise in COVID-19 cases, respectively, and also linked these pollutants to increases in severe COVID-19 cases.
Lu et al., 2021; China [35]	Cross-Sectional	2020	PM2.5, O3, SO2, NO2	COVID-19	The highest cumulative risk ratios (RR) showcase that PM2.5 at lag 0−14, O3 at lag 0−1, and SO2 at lag 0 are associated with increased risks, while SO2 indicates the highest cumulative RR (1.213), followed by O3 (1.080) and PM2.5 (1.016).
Laxmipriya and Narayanan 2021; India [36]	Cross-Sectional	–	PM 10, PM 2.5	COVID-19	Areas with limited pollution concentration have fewer COVID cases, which consist of five, resulting in a healthier population less vulnerable to COVID-19 infections. On the other hand in highly polluted areas, the cases account for over 90 % of the total proving that individuals in these areas are more prone to rapid infections.
Sangkham et al. 2021; Thailand [37]	Cross-Sectional	2020	PM10, PM2.5	COVID-19	The negative Spearman and Kendall rank correlation coefficients indicate a moderate inverse relationship between PM2.5 and PM10 levels and daily confirmed COVID-19 cases, with PM10 showing a stronger inverse correlation than PM2.5 across both correlation methods.
Kutralam-Muniasamy et al. 2021; Mexico [38]	Cross-Sectional	2020	PM10, PM2.5	COVID-19	The data suggests moderate positive correlations between PM2.5 and both daily confirmed COVID-19 cases (0.33) and deaths (0.30), and stronger correlations between PM10 and daily confirmed cases (0.53) and deaths (0.50), indicating a potential link between higher particulate matter levels and increased COVID-19 severity.
Sahoo 2021; India [39]	Cross-Sectional	2020	PM2.5, PM10, NO2, SO2	COVID-19	The study identifies air pollutants such as PM10, PM2.5, and NO2 and meteorological factors such as temperature significantly associated with COVID-19 cases. However, SO2 and humidity were found negatively associated
Mehmood et al., 2021; Pakistan [40]	Cross-Sectional	2020	PM2.5	COVID-19	A weak positive correlation is shown between PM2.5 concentration and COVID-19-infected cases in Peshawar (0.08), and Lahore (0.02), while Karachi (−0.27), and Islamabad (−0.16) exhibits weak negative associations between air pollutants and COVID-19 cases in other cities in Pakistan
Páez-Osuna, Valencia-Castañeda, and Rebolledo 2022; Mexico [41]	Cross-Sectional	2020–2021	PM2.5	COVID-19	The improved adjustment using PM2.5 emissions and cumulative mortality rates presented a positive logarithmic association; for vehicle-emitted PM2.5, it goes \(y = 81.031 \log x + 71.062 \) (r = 0.616, p < 0.005), and for anthropogenic sources, it was \(y = 111.826 \log x - 42.900 \) (r = 0.746, p < 0.005).
Sherris et al. 2021; Bangladesh [42]	Observational	2005–2014	PM2.5		The RR reported in the result was 1.032 for pneumonia.
Samillan et al. 2021; Peru [43]	Observational	2020	PM2.5, NO2	Pneumonia	A positive correlation between total infections with PM2.5 is shown through correlation analysis. Mean NO2 was the most influential factor for mortality for individuals.
Nor et al., 2021; Malaysia [44]	Cross-Sectional	2020	PM2.5	COVID-19	Despite the decreased amount of PM2.5 levels, SARS_CoV-2 was still detected mostly due to viral shedding from patients with symptoms in a poorly ventilated space with prolonged air sampling enhancing the detection.
Wannaz et al. 2021; Peru [45]	Time-series analysis	2019–2020	PM2.5, PM10	COVID-19	PM2.5 and COVID-19 Cases have positive correlation R^2^ = 0.21 (p = 0.0093). PM10 and COVID-19 Cases have positive correlation R^2^ = 0.18 (p = 0.0255).
Ma et al., 2021; China [46]	Observational	2020	PM2.5, PM10, CO, O3, NO2, SO2	COVID-19	The cumulative Risk Ratios (lag 0–7) between Tave, THI, and K and daily confirmed COVID-19 cases were 0.953, 0.982, and 0.984.
Priyankara et al., 2021; Sri Lanka [47]	Observational	2019	PM2.5, PM10	Respiratory Diseases	PM2.5 was related to an increased risk of respiratory disease hospitalization by 1.95 % PM10 was associated with 1.63 %, PM2.5 or asthma hospitalizations were 4.67 % and PM10 was 4.04 %.
Meo et al., 2022; India [48]	Observational	2020–2021	PM2.5, CO, O3, NO2	COVID-19	A one-unit increase in CO, O3, and NO2 levels, the number of cases was significantly raised by 0.77 %, 0.45 %, and 4.33 %.
Liu, Xu, and Lu 2021; China [49]	Cross-Sectional	2013–2017	SO2, NO2, CO, O3, PM2.5	Tuberculosis	Each 1 μg/m^3^ increase in SO2 at lag 0–180 days is associated with a 1.33 % increase in new TB infections, while each 1 μg/m^3^ increase in PM2.5 at lag 0–365 days is linked to a 3.04 % increase in new TB infections.
Vasquez-Apestegui et al. 2021; Peru [50]	Cross-Sectional	2020	PM2.5	COVID-19	With each unit increase in PM2.5 exposure, the number of COVID-19 deaths increases by 0.0014 units. The case fatality rate was 1.93 %.
Sangkham et al. 2023; Thailand [51]	Cross-Sectional	2020–2021	PM2.5	COVID-19	The influence of summer and rainfall, especially seasons influence raising the number of COVID-19 cases with an adjusted R-square of 0.852 and 85.60 % of the variance explained and significant association (p < 0.05) with PM2.5.
X. Wang et al. 2021; China [52]	Observational	2016–2018	PM1, PM2.5, PM10	Pneumonia	For each 10 μg/m^3^ increase, the risk of pneumonia increased by 10.28 % for PM1, 1.21 % for PM2.5, and 1.10 % for PM10.
Ali et al., 2022; Afghanistan, Bangladesh, China, India, Iran, Iraq, Pakistan [53]	Cross-Sectional	2019–2020	NO2	COVID-19	A significant positive (P < 0.05, 95 % confidence interval, two-tailed) correlation between nitrogen dioxide concentration and changes in the aerosol index for the net active-COVID cases.
Popovic et al., 2023; China [54]	Cross-Sectional	2005–2017	PM2·5, NO2, O3	TB	PM2.5 exposure over the preceding three years is highly associated with a 35 % higher TB notification rate (IRR: 1.35, 95 % CI: 1.25–1.48), while a 4 ppb rise in NO2 exposure is associated with a 25 % higher TB notification rate in the crude model (IRR: 1.25, 95 % CI: 1.08–1.44), which indicates a clear connection between higher pollution concentration and increased TB incidence.
Meo et al. 2022; Pakistan [55]	Cross-Sectional	2020–2021	PM2.5	COVID-19	In Karachi, Lahore, and Islamabad, the study revealed a significant relationship between COVID-19 instances and results. Nonetheless, the correlation was noticeable in Lahore but not in Karachi or Islamabad. Compared to Islamabad, Karachi had 2.86 times fewer COVID-19 cases. Lahore had 1.4 times as many, while Karachi had 3.6 times more fatalities and Lahore had 2.8 times as much.
Huihui Zhang et al., 2022; China [56]	Observational	2014–2017	PM2.5, SO2, NO2	Pneumonia	PM2.5 had a maximum RR of 1.198 at lag 0–11, SO2 had 1.304 at lag 0–13, and NO2 had 1.286 at lag 0–14.
Xiao et al. 2022; China [57]	Observational	2013–2019	PM2.5	ALRIs	The daily mean PM2.5 levels are associated with 11.20 % ERs, regular exceedance concentration hours (DECH) with a 12.30 % ER, and hourly peak PM2.5 levels with a 9.73 % ER, highlighting increased health risks as exposure intensity or duration increases.
Khalis et al. 2022; Morocco [58]	Observational	2020	SO2, NO2, O3, CO, PM10	COVID-19	The correlations indicate that the pollutants CO (0.58), NO2 (0.60), O3 (0.42), PM10 (0.40), and SO2 (0.46) had a slightly positive relation with daily COVID-19 instances, with NO2 exhibiting the highest effect. The correlations indicate that the pollutants CO (0.58), NO2 (0.60), O3 (0.42), PM10 (0.40), and SO2 (0.46) had a somewhat positive link with daily COVID-19 instances, with NO2 exhibiting the highest effect.
Khan, 2022; Pakistan [59]	Cross-Sectional	–	NO2, PM10, PM2.5	COVID-19	A significant positive (P < 0.05, 95 % confidence interval, two-tailed) correlation between nitrogen dioxide concentration and changes in the aerosol index for the net active-COVID cases.
Jainonthee et al. 2022; Thailand [60]	Cross-Sectional	2011–2020	PM2.5, PM 10	Pneumonia	The incidence rate of pneumonia was the highest in 2018 (697.8 per 100,000 population) while calculated as the lowest in 2020 (404.6 per 100,000 population).
Gonçalves et al. 2023; Brazil [61]	Cross-Sectional	2020–2021	PM2.5	COVID-19	The relative risks (RR) for COVID-19 notified cases are 1.8 in Amazon, 1.5 in Acre, 1.8 in Para, 1.6 in Mato Grosso, and 1.4 in Rondonia.
Damasceno et al. 2022; Brazil [62]	Observational	2020–2021	PM2.5, NO2, O3	COVID-19	1 g/m3 increase in the long-term average of PM2.5 was in correlation with a 10.22 %.
Y. Zheng et al., 2023; China [63]	Cross-Sectional	2017–2019.	PM2.5 PM2.5-bound As, Cd, Co, Cr (VI), Ni, Pb	ALRIs	A 10 μg/m^3^ increase in the pollutants is associated with a 2.89 % increase in respiratory diseases, a 2.74 % increase in AURIs, a 23.36 % increase in influenza and pneumonia, and a 16.86 % increase in ALRIs.
Bonilla et al., 2023; Brazil, Colombia, and Mexico [64]	Cross-Sectional	2020	N/A	COVID-19	In COVID-19 mortality rates, Prolonged exposure to an increase of 1 μg/m^3^ in fine particles results in a 2.7 % increase.
Brazil, Colombia, and Mexico [64]					
Q. Wang et al. 2023; China [65]	Cross-Sectional	2004–2018	PM2.5, PM10, SO2, NO2, CO, O3.	TB	South China has a median monthly relative humidity of 78.76 %, rainfall of 118.22 mm, and temperature of 24.11 °C, while North China has the highest median monthly SO2 levels at 24 μg/m^3^ and NO2 at 40.5 μg/m^3^.
Chen et al., 2023; China [66]	Time-series analysis	2005–2018	NO2, PM2.5, PM10	Influenza	The age 15–24 group shows an increase in the relative risk of influenza with an increase in ¬PM2.5 concentration, with a lag of 0–6 months (ERR 1.08, 95 % CI 0.10–2.07).
Nie et al., 2023; China [67]	Cross-Sectional	2018–2021	PM2.5, PM10, NO2, and SO2	TB, URTI, pneumonia, COPD	The average mass concentration of PM2.5, PM10, NO2, and SO2 in ambient air was associated with an elevated incidence of respiratory diseases by 0.2–1.4 %, 0.7–1.6 %, 3.7–8.2 %, and 0.5–2.3 %, respectively; however, a monthly mean mass level of CO increased by 1 mg/m3 led to an increase in pulmonary tuberculosis incidence by 2.9 %.
Luo et al., 2024; China [68]	Longitudinal	2010–2021	PM2.5, SO2	TB	If the average mass concentrations of PM2.5, PM10, NO2, and SO2 increase in ambient air, it associates with a rise in respiratory diseases by 0.2–1.4 %, 0.7–1.6 %, 3.7–8.2 %, and 0.5–2.3 %, respectively, while a 1 mg/m^3^ increase in CO levels led to a 2.9 % rise in pulmonary tuberculosis incidence, with a 1-unit increase in PM2.5 raising the notification rate by 0.396 %, and relative risks (RR) for respiratory diseases varying by clusters.

∗(--) data was not available.

## 3 Results

### 3.1 Search and selection

**Fig. 1:**
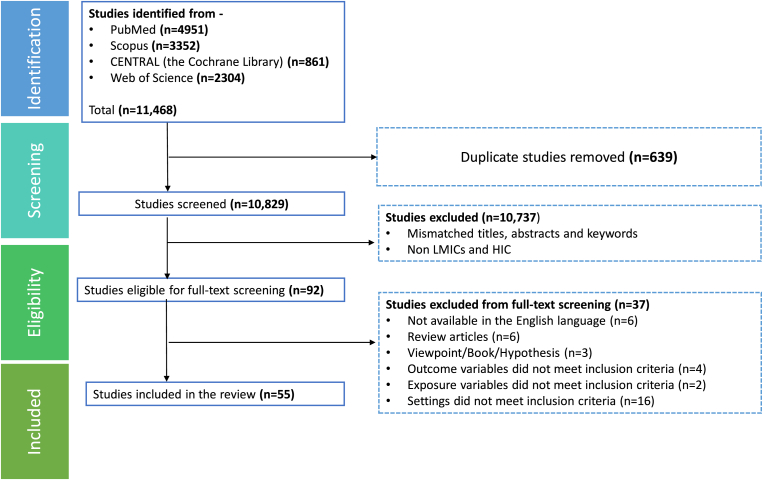
Flow diagram of study selection.

A total of 11,468 studies were identified from four electronic databases. Among these, 639 duplicate records were removed from all databases. After removing duplicates, the titles and abstracts of 10,829 articles were screened, from which 92 full-text articles were reviewed. Finally, 55 articles were included in the synthesis (Fig. 1). A total of 37 studies were excluded for not meeting the eligibility criteria (Table S3), including reasons such as non-English language (n = 6), review articles (n = 6), viewpoint, hypotheses, books (n = 3), mismatch of outcome (n = 4) or exposure variables (n = 2) with our inclusion criteria, and inappropriate setting (n = 16) such as country or population.

### 3.2 Study design and study period

**Figure_2:**
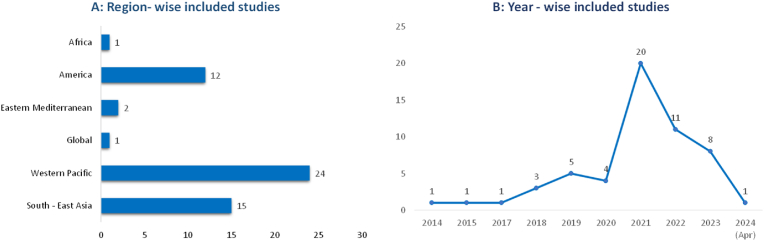
Distribution of the included studies by region (A) and publication period (B).

The design of the studies reviewed was varied. Among the 55 studies, 36 were cross-sectional, making up the majority. Additionally, 11 observational studies, 3 case-control studies, 2 longitudinal and 3 time- series studies were included. The review also noted that 28 studies failed to report sample size, which can affect the interpretability of results. Most of the studies (n = 40) were conducted since 2020 (Fig. 2B), likely influenced by the onset of the COVID-19 pandemic, while the rest (n = 15) were conducted before this period. A few (n = 4) studies did not report the timeframe of their research.

### 3.3 Examined geographical areas

The reviewed studies cover a wide geographical distribution (Fig. 2A) defined by the World Health Organization (WHO) [14]. The largest portion (n = 24) originated from China in the Western Pacific Region, followed by studies from the Americas (n = 12). Despite the heightened vulnerability of the African region to both infectious diseases and pollution, only one study from this region, conducted in Benin was included. Southeast Asian countries (n = 15) such as India and Bangladesh which experience high levels of air pollution had moderate representation (Fig. 2A). We also found one multi-country/global study that mostly focuses on LMICS and South Asian countries.

### 3.4 Summary of risk of bias assessment

**Figure_3:**
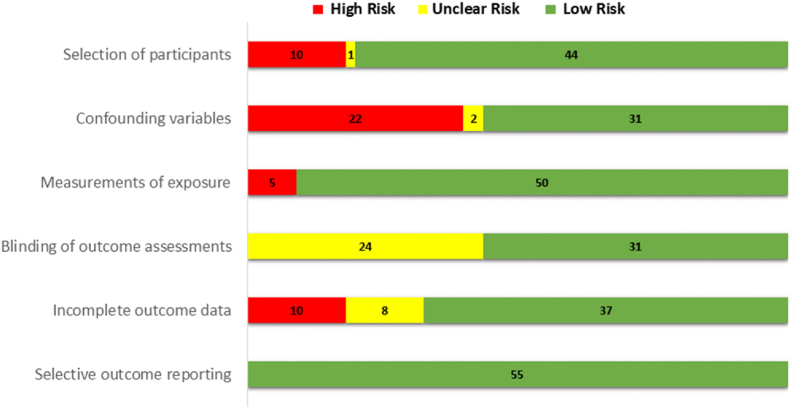
Summary of risk of bias assessment within included studies.

Out of the 55 studies, 44 were assessed as having a low risk of bias, 10 as high risk and 1 as unclear risk, indicating that the selection process for most studies was robust and minimally biased (Fig. 3; Table S5). ‘The confounding’ variable domain with the highest proportion of high-risk bias was identified at 22 studies with an additional 2 studies being unclear and 31 studies classified as low risk of bias. This suggests a potential for confounding factors to affect study outcomes across several studies. In the ‘Measurements of the Exposure domain’, the risk of bias was predominantly low, with 5 studies classified as low risk. However 5 were considered high risk, indicating concerns regarding exposure measurement accuracy. No studies were assessed as having an unclear risk. For ‘the blinding of outcome assessments’, 24 studies had an unclear risk of bias, indicating insufficient information. However, none were deemed high risk, and 31 studies were found to have a low risk, reflecting adequate blinding in a considerable proportion of the studies. Incomplete outcome reporting was deemed reliable in the majority of studies, with 37 studies rated as low risk. However, 10 studies were considered high risk and 8 studies were unclear suggesting potential issues with the completeness of outcome data in some studies. Finally, selective outcome reporting was rated predominantly as low risk, with all the studies showing no concerns for selective reporting. Notably, no studies were identified as high or unclear risk in this domain, indicating consistent and transparent reporting across most studies.

Overall, the risk of bias assessment indicates a generally favourable profile across the domains, particularly in the areas of selection of participants, measurements of exposure and selective outcome reporting where most studies are classified as low risk. However, potential biases in confounding variables and blinding of outcome assessments were identified as areas requiring careful consideration when interpreting the results of the included studies.

### 3.5 Result assessment and summarization

The majority of the studies primarily examine the relationship between air pollutants and infectious diseases, with a significant emphasis on COVID-19. The most frequently studied pollutants were PM2.5 (particulate matter with a diameter of 2.5 μm or less) and PM10 (particulate matter with a diameter of 10 μm or less), both known for their ability to penetrate deep into the lungs and cause inflammation. Other pollutants include CO (carbon monoxide), O3 (ozone), NO2 (nitrogen dioxide), SO2_2_ (sulfur dioxide), and heavy metals such as Pb, Cd, Ni (nickel), and Cr (chromium), which are commonly associated with urban and industrial pollution. These exposures were studied across various regions, highlighting the global health impact of poor air quality, particularly in exacerbating respiratory diseases and increasing susceptibility to infections like Coronavirus disease (COVID-19). COVID-19 was the most commonly researched infectious disease, followed by tuberculosis (Table 1). However, we were unable to find any eligible studies examining the association with certain widely distributed environmental toxicants, such as persistent organic pollutants (POPs), phthalates, bisphenol A or polycyclic aromatic hydrocarbons (PAHs).

#### 3.5.1 COVID-19

##### 3.5.1.1 Particulate matter and outcomes

Twenty-five studies reported significant effects of particulate matter (PM2.5 and PM10) on COVID-19, including increased incidence, spread, morbidity, and mortality (Table S4). Most studies focused on regional variations in infection rates, while others examined clinical outcomes such as hospitalization and death. For instance, in China, prolonged exposure to PM2.5 was linked to a 2.7% increase in COVID-19 mortality in high-risk areas. Several studies demonstrated positive associations across cities in Pakistan, where COVID-19 incidence was significantly higher in Karachi (0.004, p = 0.000), Islamabad (0.003, p = 0.023), and Lahore (0.003, p = 0.000), with NO_2_ levels identified as a key driver [48]. Mechanistically, fine particulate matter (≤2.5 μm) may adsorb SARS-CoV-2 virions, prolong aerosol viability and facilitate deeper respiratory penetration, intensifying infection severity. However, studies such as Zhu et al., ^33^and Liu et al., [69] presented a high or unclear risk of bias due to inadequate confounding control and lack of outcome blinding.

##### 3.5.1.2 Gaseous pollutants and COVID-19 transmission

Several studies examined the association between gaseous pollutants such as nitrogen dioxide (NO_2_), sulfur dioxide (SO_2_), carbon monoxide (CO), and ozone (O_3_) and COVID-19 outcomes [48]. reported that a unit increase in O_3_, CO, and NO_2_ was associated with respective increases in COVID-19 cases by 0.45%, 0.77%, and 4.33%. These pollutants may impair pulmonary immune defences and epithelial barrier integrity, promoting viral transmission and worsening disease severity. Across multiple geographies, gaseous pollutants were consistently associated with elevated COVID-19 incidence and mortality, further supporting their role as risk enhancers during the pandemic. Chronic exposure to these particulates also upregulates pulmonary ACE2 receptor expression, thereby enhancing viral entry and initial replication in epithelial cells [70]. Concurrently, gaseous irritants such as NO_2_ and O_3_ disrupt epithelial barrier integrity and trigger excessive release of pro-inflammatory cytokines (e.g. IL-6, TNF-α), priming the lungs for exaggerated inflammatory responses upon infection [71].

#### 3.5.2 Tuberculosis

##### 3.5.2.1 Particulate matter (PM2.5 and PM10)

Five studies explored the association between tuberculosis (TB) and exposure to fine and coarse particulate matter , reporting a significant positive correlation. TB remains a major concern in regions with high industrial emissions and dense urban populations, where air quality is often poor. One study [25] demonstrated that a 1 μg/m^3^ increase in PM2.5 was associated with a 3.04% increase in new TB infections, highlighting a strong correlation. Chronic exposure to PM2.5 and PM10 induces oxidative stress in lung tissues, promotes apoptosis of alveolar macrophages and impairs phagocytic clearance of *Mycobacterium tuberculosis*. Mechanistically, particulate matter exposure leads to a dysregulated immune response characterized by elevated pro-inflammatory cytokines (TNF-α and IL-1β), which disrupt granuloma formation and facilitate bacterial replication. This contributes to both new TB infections and potential reactivation of latent TB.

##### 3.5.2.2 Gaseous pollutants (SO_2_, NO_2_, CO)

Three studies also assessed the contribution of gaseous pollutants to TB outcomes, with varying degrees of association: SO_2_ was found to be moderately associated with TB incidence, with each 1 μg/m^3^ increase linked to a 1.33% rise in infections [25]; NO_2_ showed a weaker but still positive association; CO exposure was particularly impactful [67]; reported a 2.9% increase in TB incidence per 1mg/m^3^ monthly rise in CO concentration.

#### 3.5.3 Upper and lower respiratory tract infections

##### 3.5.3.1 Particulate matter (PM2.5 and PM10)

Five studies investigated the association between particulate matter (PM2.5 and PM10) and pneumonia incidence. All reported a positive correlation with higher concentrations of PM linked to increased pneumonia cases (Table S4). The highest inceidence of pneumonia was reported in Thiland in 2018 (697.8 per 100,000) whichdeclining to 404.6 per 100,000 in 2020, likely reflecting reduced airborne pollutants emissions during COVID-19 lockdowns.[60]. PM2.5 and PM10 compromise mucociliary clearance, impair epithelial antimicrobial defence and promote alveolar macrophage dysfunction [22]. This leads to increased vulnerability to bacterial and viral respiratory infections. Mokoena et al., 2019 [22] also exhibited a high risk of bias due to issues with how exposure was measured and unclear blinding of outcomes, which may lead to uncertainties in quantifying the true magnitude of the impact of air pollutants on pneumonia incidence. Additionally, PM-induced mitochondrial damage and reactive oxygen species (ROS) generation contribute to chronic pulmonary inflammation.

In studies examining acute lower respiratory infections (ALRIs), PM2.5 containing transition metals such as nickel chromiumCr, and arsenic was strongly associated with a 16% increase in ALRI risk per 10 μg/m^3^ increase in concentration [72]. The metal-laden particulates promote oxidative injury, activate NF-κB signalling, and impair immune cell bactericidal functions leading to severe infections such as pyothorax and lung abscesses particularly in children. In addition to pneumonia and ALRIs, four studies reported a positive association between PM2.5 exposure and influenza infection (Table S4). A long-term study in Beijing (2008–2011) found that increases in PM2.5 were associated with a lag of up to two months in influenza onset, suggesting a delayed but significant effect of air pollution on viral transmission [73] also Liang et al., 2014 included in the review had unclear risk of bias in multiple domains, showing an interpretation of the temporal associations.

Mechanistically, PM2.5 can adsorb influenza virions, prolong their aerosol viability, and enable deeper respiratory tract deposition [74]. These particulates also induce epithelial damage and generate ROS, which suppress interferon responses (e.g., IFN-α, IFN-β) and mucosal immunity leading to delayed viral clearance and heightened infection risk [15].

##### 3.5.3.2 Gaseous pollutants (O_3_, SO_2_, NO_2_, CO)

Several studies also identified strong associations between gaseous pollutants and respiratory tract infections. Mokoena et al. (2019) found increased pneumonia risk associated with multiple gases: PM2.5 (OR = 1.328), O_3_ (OR = 2.061) and SO_2_ (OR = 1.524) [22]. These pollutants contribute to airway inflammation, epithelial barrier disruption, and impaired host immune response, establishing conditions that promote respiratory infections.

##### 3.5.3.3 Heavy metals (Ni, Cr, As)

The toxic metal content of PM2.5, specifically Ni, Cr and As has been shown to significantly increase the risk of severe ALRIs. These metals drive oxidative stress, compromise mucosal immunity, and heighten vascular permeability, predisposing individuals particularly children to invasive lung infections.

#### 3.5.4 Other respiratory infections

##### 3.5.4.1 Particulate matter (PM2.5 and PM10)

While most studies focus on respiratory outcomes, a limited number have explored the broader systemic effects of particulate matter on infectious diseases. Findings from Brazil indicated a positive association between PM2.5 exposure and severe acute respiratory disease (SARD), with observed changes in infection dynamics across different regions [28] (Table S4). Although evidence is still emerging, these results suggest that chronic PM exposure may contribute to the progression or severity of infectious diseases beyond the respiratory tract.

##### 3.5.4.2 Gaseous pollutants (NO_2_, SO_2_, CO)

Airborne gaseous pollutants such as nitrogen dioxide, sulfur dioxide, and carbon monoxide have also been linked to systemic infectious outcomes. While not as extensively studied in the context of diseases like AIDS or hepatitis C, these pollutants are known to induce oxidative stress, epithelial dysfunction and chronic inflammation, which may indirectly impact immune function and susceptibility to severe infectious diseases. The contribution of gaseous pollutants to non-respiratory infections remains an area in need of further investigation.

##### 3.5.4.3 Heavy metals (Pb, Cd, Hg, fe, Co, Mn, Cu)

Two studies provided insights into the relationship between heavy metal exposure and chronic infectious diseases such as AIDS and Hepatitis C. However, a high risk of bias was reported in areas such as confounding control and exposure assessment. Elevated concentrations of lead, cadmium and mercury were detected in the blood of AIDS patients compared to healthy controls. Similarly, individuals with Hepatitis C showed significantly increased levels of iron, cobalt manganese and copper. These elevated metal burdens suggest that environmental exposure may exacerbate the clinical condition of chronically infected individuals by impairing immune regulation and contributing to disease progression. Mechanistically, chronic heavy metal exposure disrupts lymphocyte proliferation, reduces CD4^+^ T-cell counts, and impairs antibody production, contributing to immunosuppression in vulnerable populations [28]. In individuals living with AIDS or hepatitis C, such immune dysfunction may reduce the body's ability to control viral replication and eliminate infected cells, worsening long-term health outcomes.

## 4 Discussion

This systematic review examined the relationship between environmental toxicants, specifically air pollutants and infectious diseases focusing on LMICs. The findings highlight the significant association between exposure to particulate matter and an increased risk of respiratory infections such as COVID-19, tuberculosis, acute lower respiratory infections and pneumonia. Additionally, heavy metals were implicated in chronic infectious diseases like hepatitis and HIV/AIDS. Although these associations were consistently observed across the studies, the underlying mechanisms remain partially understood. Evidence generated so far suggest exposure to toxic agents may lead to immunotoxic changes in hosts that disturb immunological balance in the host, reduce the threshold for preventing or combating infections, increase the rate of pathogen shedding or changes in pattern, extend the length of infections and multiply the severity [75,76].

The biological mechanisms underlying these associations remain partially understood but point to immunotoxic effects that compromise host defences. Exposure to environmental toxicants may alter immune system function, reduce resistance to infections, increase the duration and severity of illness, and enhance pathogen shedding or transmission patterns. These mechanisms are captured in a conceptual model proposed by Feingold et al. (2010) [75], which illustrates how toxicants and infectious agents can act synergistically. Toxicants may disrupt immune regulation or tissue integrity, facilitating pathogen replication and exacerbating infection outcomes. Traditional susceptibility factors such as genetics, age, and nutritional status may further modulate these effects, influencing whether outcomes range from recovery to chronic illness or mortality. Environmental media such as air, water, food, soil, and dust serve as shared pathways for toxicants and infectious agents, often stemming from common sources and vectors. Once inside the body, toxicants may accumulate to biologically active levels that disrupt immune function, while pathogens exploit these compromised defences to establish and sustain infection.

Our findings are also aligned with the World Health Organization's Environmental Health Indicators Framework, particularly the DPSEEA model (Driving Force–Pressure–State–Exposure–Effect–Action), which links environmental determinants to health outcomes [77]. This framework emphasizes the role of systemic and structural environmental pressures in shaping health vulnerabilities in LMICs and supports the need for integrated strategies to mitigate both toxicant exposure and infectious disease burden.

### 4.1 The role of air quality

The majority of studies reviewed underscored the critical role of particulate matter, particularly PM2.5 in exacerbating infectious disease outcomes. PM2.5 is known for its ability to penetrate deep into the lungs and trigger inflammation, impairing the immune response and increasing susceptibility to infections. This was most evident in studies focusing on COVID-19 where higher levels of PM2.5 were consistently linked to increased incidence, morbidity, and mortality. These results are in line with research conducted in India and Italy, where regions with higher pollution levels saw a more severe spread of COVID-19 [78]. A study conducted in India where people living in areas of higher PM2.5 and PM10 pollution levels were more susceptible to COVID-19 infections than people living in areas of low pollution. Liu et al., also conducted research in nine countries using air pollution data and reported COVID-19 incidence, highlighting that high air pollution concentration accompanied by a decline in air quality causes an increase in newly confirmed cases of COVID-19 infection [69]. One of the dominant sources of PM2.5 is household air pollution (HAP) globally and HAP contributes 30% of PM2.5 exposure in South Asia [79]. Bates et al., investigated indoor air pollution sources finding a high risk of PM2.5 exposures from biomass stoves although the risk from kerosene stoves was higher [80].

### 4.2 The role of oxidative stress

The role of oxidative stress in mediating the impact of PM2.5 on infectious diseases was frequently mentioned in the literature. Exposure to PM2.5 can induce oxidative stress, which in turn impairs respiratory tract defences, facilitating the onset and progression of infections such as tuberculosis. Specifically, tuberculosis-infected lung tissues exposed to high PM2.5 levels demonstrated decreased cell viability and reduced production of antimicrobial peptides further exacerbating disease progression. Many studies indicate that oxidative stress is responsible for acquiring infectious diseases [81,82]. Exposure to PM2.5 and PM10 impairs the immune system of the respiratory tract, causing oxidative stress and down-regulating the immune response to tuberculosis, increasing the host's susceptibility to infection and further worsening the disease [83,84]. Tuberculosis-infected lung tissue, specifically A549 cells, showed a statistically significant decrease in viability at exposure to high concentrations of PM2.5 and PM10. This exposure reduced their ability to produce antimicrobial peptides upon infection. Among the two, PM2.5 has been shown to induce greater toxicity than PM10, likely due to its smaller size, deeper pulmonary penetration and higher content of toxic constituents [83].

### 4.3 Impacts of heavy metal exposure on chronic infectious diseases

While respiratory infections dominated the findings, heavy metal exposure also emerged as a serious concern for individuals with chronic infectious diseases such as HIV/AIDS and hepatitis. However, the evidence on exposure levels to environmental toxicants among the HIV/AIDS population is very limited. We have found only one study from the African region that reported a high concentration of heavy metals (Pb, Cd, Hg, Cr, and Ni) among people living with HIV [19]. This result is also comparable with a study conducted by the National Health and Nutrition Examination Survey (NHANES) in the USA, which found the HIV-infected population had higher blood heavy metals levels than the control group [85]. This negatively affects the prognosis of HIV-1 infection, likely due to the combined impact of environmental exposures and chronic inflammation. In contrast, several studies have attributed the higher blood levels of toxic metals in HIV-infected individuals to impaired renal and liver functions, which limit the clearance of these metals [19]. A recent research conducted in Niger Delta of Nigeria also supports these findings [88], presenting higher levels of heavy metals in HIV/AIDS individuals and their associations with biomarkers of oxidative stress, inflammation, as well as DNA damage This highlights the vulnerability of this population to environmental toxicants in LMIC settings where environmental and regulatory protection is not well established.

### 4.4 Gaps in the literature

Despite the robust associations found between air pollution and infectious diseases, several gaps still remain. For instance, none of the studies reviewed examined the effects of persistent organic pollutants (POPs), phthalates, bisphenol A, or polycyclic aromatic hydrocarbons (PAHs) on infectious diseases despite their well-documented health risks, particularly related to chronic noncommunicable diseases such as the increased risk of cancer, cardiovascular diseases [86]. Future research should address these gaps by exploring the broader range of environmental toxicants and their potential interactions with infectious pathogens which is a relatively new concept [1,75]. Moreover, while PM2.5 and PM10 were the most frequently studied pollutants, there was limited analysis of other common urban pollutant gases such as nitrogen dioxide and ozone. These pollutants are also known to contribute to respiratory morbidity and their effects on infectious diseases warrant further investigation.

## 5 Strengths and limitations of the current evidence base

This systematic review synthesizes existing evidence on the relationship between environmental toxicant exposure and infectious disease outcomes in LMICs, contributing to a better understanding of how environmental factors may influence disease dynamics in vulnerable settings. While progress has been made in understanding these relationships, further research is needed to elucidate the specific genomic mechanisms at play and to address the existing gaps in the literatures. Other strengths include the unrestricted publication dates for articles and a standard robust approach to systematic review. However, this study is not without limitations. First, all non-English studies were excluded from this systematic review and therefore, we may have missed important findings in other languages. However, articles excluded for languages were only six in number. Another limitation is that due to the inconsistencies in the types and number of study populations, location of different toxicant exposure measures, and differing outcomes, we were unable to conduct a meta-analysis. Similarly, we are unable to assess the publication bias as a meta-analysis was not done. However, we have used a comprehensive search study and databases, we believe we have not missed any potential articles. Also, the review would benefit from a more in-depth analysis of confounding variables. Some results are described with p-values and correlation coefficients, but not all findings include confidence intervals. It is important to ensure that all statistical findings are presented with 95% confidence intervals where applicable to provide a sense of the precision of the estimates. The search terms might not have been all-inclusive as toxicants might have been classified differently. Lastly, to ensure the quality of the papers, this systematic review only focused on studies published in peer-reviewed journals; nevertheless, other sources may also contain significant and pertinent information.

## 6 Implications for public health policy and practices

Growing evidence indicates that a wide array of environmental pollutants in LMICs, including fine particulate matter (PM2.5 and PM10), gaseous pollutants (NO2, SO2, CO, O3), and toxic heavy metals (Pb, Cd, Ni, Cr, As) are associated with increased infectious disease risks [87] specially after COVID-19 pandemic. These exposures contribute substantially to the disease burden in LMIC populations: over 90% of pollution-related deaths occur in low- and middle-income countries [1] with air pollution alone linked to a large fraction of respiratory infection mortality [43]. However, LMICs face unique challenges such as weak regulatory and monitoring capacity, high urban population density, and limited healthcare infrastructure [44]. Addressing these gaps requires urgent, evidence-based public health policies tailored to LMIC contexts. For example, strengthening air quality monitoring networks is crucial for data-driven action, as many cities lack reliable pollution surveillance [45]. Implementing stricter emissions controls on traffic, industrial and residential sources coupled with cleaner fuels and technologies can reduce ambient particulate and noxious gas levels, helping to meet WHO air quality targets [87]. Improved urban planning (e.g. better waste management and regulated industrial siting) is also needed to limit pollutant exposure in densely populated areas, while integrating environmental indicators into infectious disease surveillance can enable early detection of pollution-related health trends [46]. Major international agencies such as WHO and UNEP (United Nations Environment Programme) have accordingly urged multisectoral collaboration, stronger environmental legislation and 'One Health' approaches to jointly tackle pollution and infectious disease burdens [47]. Likewise, the World Bank has emphasised making pollution control a development priority in LMICs. Adoption of these policies in resource-limited settings could significantly reduce pollutant exposures and infection risks, while yielding co-benefits for climate change mitigation and overall public health.

## 7 Recommendation for future research

To strengthen the evidence base, future studies would benefit from improved methodologies and collaboration as well as prioritising research on both classic (PM2.5, NO_2_, SO_2_) and understudied pollutants (POPs, PAHs, BPA, phthalates) in relation to a wider array of infections (beyond COVID-19 and TB to include parasitic, diarrhoeal and zoonotic diseases), for example.

• **Longitudinal study designs:** Emphasising cohort and long-term follow-up studies can establish temporal links and capture the cumulative effects of chronic exposures on infection risk (long-term pollution exposure often has a more marked health impact than short-term exposure.)
• **Individual-level exposure data:** Greater use of personal exposure monitoring and epigenetic analysis is needed to improve exposure assessment. Collecting high-resolution individual data (rather than only ambient averages) will reduce misclassification and enable more precise exposure–response analyses.
• **Integrated surveillance and One Health approach:** Linking environmental monitoring data with health surveillance (disease incidence) data can facilitate comprehensive analyses of pollutant–disease trends. Closer interdisciplinary collaboration between environmental scientists, epidemiologists and infectious disease experts is needed to adopt an One Health approach, recognizing the connected nature of environmental and infectious health threats.
• **Multi-pollutant models:** Investigating multiple pollutants simultaneously (instead of isolated single-pollutant analyses) is crucial to reflect real-world exposure mixtures and interactions. Complex co-exposures may have synergistic effects on infectious disease outcomes – for example, combined exposure to metals and other chemicals has been linked to increased antibiotic-resistant infections.
• **Focus onemergingpollutants:** Future research should explore the impacts of under-studied pollutant classes (e.g. persistent organic pollutants (POPs), phthalates, bisphenol A (BPA), polycyclic aromatic hydrocarbons (PAHs)) on infectious disease outcomes.
• **Infectious disease target:** Future research should target a broader range of infectious diseases beyond the current emphasis on COVID-19 and tuberculosis, including high-burden infections that have received limited attention.
• **Research capacity and collaboration in LMICs:** Building research infrastructure and expertise within LMICs is essential for sustainable progress. This includes training local researchers, establishing regional research networks, and fostering international research programmes to support advanced study designs and analytical techniques. Strengthening capacity will enable LMIC-led investigations such as community-based longitudinal cohorts and multi-centre studies, and ensure that pollution health insights are rooted in local data and context.
• **Interest holder engagement:** Global action against pollution-driven infections requires coordination among multiple interest holders including international bodies such as the WHO, UNEP and the World Bank set air-quality guidelines; national governments—through ministries of health and environmental protection agencies must integrate pollution surveillance into disease control plans and enforce emissions standards; healthcare networks and research institutes, academia should incorporate environmental risk assessment into clinical guidelines and primary care; and community organisations, NGOs and citizen-science groups mobilize grassroots monitoring, raise public awareness and advocate for stronger local enforcement.

## 8 Conclusion

This systematic review examines the critical intersection between environmental pollution and infectious disease outcomes in LMICs. The causes of infectious diseases are multifactorial and most cases occur due to a complex interplay of the immune system including immune suppression, environmental toxicants and other host factors. Many studies over the last few decades have explored the association of exposures to air pollutants, environmental heavy metals as well as other environmental chemical contaminants with inflammatory changes and several respiratory infectious diseases. Given the significance of this topic for public health, it is important to emphasize that modern pollution is one of the causes of infectious diseases. This review highlighted these two domains and highlights prospects for enhancing public health through collaborative interdisciplinary studies.

## Ethical approval

The study adhered to the Declaration of Helsinki and received approval from the Ethical Review Committee and Institutional Review Board at icddr,b in Bangladesh under the protocol number: 22030.

## Funding source

The Thrasher Research Fund partially supported the study (Award number- 01477). Rehnuma Haque is supported by the Global Health Equity Scholars Program 10.13039/100000002NIH FIC and 10.13039/100000066NIEHS D43 TW010540.

## Declaration of competing interest

The authors declare that they have no known competing financial interests or personal relationships that could have appeared to influence the work reported in this paper.
