# Supplementary material for: A systematic review of the pivotal role of environmental toxicant exposure on infectious diseases in low- and middle-income countries
Source: Public Health Pract (Oxf). 2025 Jun 25;10:100631. doi: 10.1016/j.puhip.2025.100631 (PMC12274766; doi:10.1016/j.puhip.2025.100631)
Supplement: Multimedia component 3 [file mmc3.docx]

# **Table_S3: Studies excluded after full-text review (n=37)**

|  |  |
| --- | --- |
| **Author, Year** | **Reason for Exclusion** |
| (Kalmykova & Popova, 1994) | Not available in the English language |
| (Khauadamova & Kumisbaeva, 1997) | Not available in the English language |
| (Repetto & Baliga, 1997) | Review Article |
| (Bruce et al., 2000) | Review Article |
| (Azmi et al., 2006) | Outcome variables did not meet the inclusion criteria |
| (Onishchenko et al., 2006) | Not available in the English language |
| (Bennion et al., 2007) | Exposure variables did not meet the inclusion criteria |
| (Ion-Nedelcu et al., 2008) | Not available in the English language |
| (Golub & Strukova, 2008) | Settings did not meet the inclusion criteria |
| (Shamsiyev & Khusinova, 2008) | Not available in the English language |
| (Burtseva et al., 2009) | Not available in the English language |
| (Memish et al., 2014) | Outcome variables did not meet the inclusion criteria |
| (Thapa & Pant, 2014) | Viewpoint |
| (Kumar & Tiwari, 2019) | Book |
| (Sohn et al., 2019) | Settings did not meet the inclusion criteria |
| (Reyes & Medina, 2020) | Hypothesis |
| (Gautam et al., 2020) | Outcome variables did not meet the inclusion criteria |
| (Chennakesavulu & Reddy, 2020) | Settings did not meet the inclusion criteria |
| (Borro et al., 2020) | Settings did not meet the inclusion criteria |
| (Hoang & Tran, 2021) | Settings did not meet the inclusion criteria |
| (Huh et al., 2020) | Settings did not meet the inclusion criteria |
| (Bălă et al., 2021) | Review Article |
| (Fernández et al., 2021) | Review Article |
| (Dales et al., 2021) | Settings did not meet the inclusion criteria |
| (Semczuk-Kaczmarek et al., 2022) | Settings did not meet the inclusion criteria |
| (Lym & Kim, 2022) | Settings did not meet the inclusion criteria |
| (Orak & Ozdemir, 2021) | Settings did not meet the inclusion criteria |
| (Toczylowski et al., 2021) | Settings did not meet the inclusion criteria |
| (Meo et al., 2021) | Review Article |
| (Meo, Almutairi, et al., 2021) | Settings did not meet the inclusion criteria |
| (Ben Maatoug et al., 2021) | Settings did not meet the inclusion criteria |
| (Liu et al., 2021) | Settings did not meet the inclusion criteria |
| (Ahmadi et al., 2021) | Exposure variables did not meet the inclusion criteria |
| (Akan, 2022) | Settings did not meet the inclusion criteria |
| (Shim et al., 2022) | Settings did not meet the inclusion criteria |
| (Rashidi et al., 2023) | Outcome variables did not meet the inclusion criteria |
| (Safo-Adu et al., 2023) | Review Article |

References:

1. Ahmadi, S., Ezeliora, C. D., Sharki, S. H., Osagie, C., Ghosh, S., Igwegbe, C. A., & Khan, N. A. (2021). Assessment of health impacts attributed to PM10 exposure during 2015–2017 in Zabol City, Iran. *International Journal of Environmental Science and Technology*. https://doi.org/10.1007/s13762-021-03587-6
2. Akan, A. P. (2022). Transmission of COVID-19 pandemic (Turkey) associated with short-term exposure of air quality and climatological parameters. *Environmental Science and Pollution Research*, *29*(27), 41695–41712. https://doi.org/10.1007/s11356-021-18403-4
3. Azmi, M. A., Naqvi, S. N. H., Azmi, M. A., & Aslam, M. (2006). Effect of pesticide residues on health and different enzyme levels in the blood of farm workers from Gadap (rural area) Karachi—Pakistan. *Chemosphere*, *64*(10), 1739–1744. https://doi.org/10.1016/j.chemosphere.2006.01.016
4. Bălă, G.-P., Râjnoveanu, R.-M., Tudorache, E., Motișan, R., & Oancea, C. (2021). Air pollution exposure—The (in)visible risk factor for respiratory diseases. *Environmental Science and Pollution Research*, *28*(16), 19615–19628. https://doi.org/10.1007/s11356-021-13208-x
5. Ben Maatoug, A., Triki, M. B., & Fazel, H. (2021). How do air pollution and meteorological parameters contribute to the spread of COVID-19 in Saudi Arabia? *Environmental Science and Pollution Research*, *28*(32), 44132–44139. https://doi.org/10.1007/s11356-021-13582-6
6. Bennion, P., Hubbard, R., O’Hara, S., Wiggs, G., Wegerdt, J., Lewis, S., Small, I., van der Meer, J., Upshur, R., & on behalf of the Medecins san Frontieres/Aral Sea Respiratory Dust and Disease project team. (2007). The impact of airborne dust on respiratory health in children living in the Aral Sea region. *International Journal of Epidemiology*, *36*(5), 1103–1110. https://doi.org/10.1093/ije/dym195
7. Borro, M., Di Girolamo, P., Gentile, G., De Luca, O., Preissner, R., Marcolongo, A., Ferracuti, S., & Simmaco, M. (2020). Evidence-Based Considerations Exploring Relations between SARS-CoV-2 Pandemic and Air Pollution: Involvement of PM2.5-Mediated Up-Regulation of the Viral Receptor ACE-2. *International Journal of Environmental Research and Public Health*, *17*(15), 5573. https://doi.org/10.3390/ijerph17155573
8. Bruce, N., Perez-Padilla, R., & Albalak, R. (2000). Indoor air pollution in developing countries: A major environmental and public health challenge. *Bulletin of the World Health Organization*, *78*(9), 1078–1092.
9. Burtseva, E. I., Shevchenko, E. S., Beliakova, N. V., Oskerko, T. A., Kolobukhina, L. V., Merkulova, L. N., Vartanian, R. V., Prilipov, A. G., Rotanov, M., & Zaplatnikov, A. L. (2009). [Monitoring of the sensitivity of epidemic influenza virus strains isolated in Russia to etiotropic chemical agents]. *Voprosy Virusologii*, *54*(5), 24–28.
10. Chennakesavulu, K., & Reddy, G. R. (2020). The effect of latitude and PM2.5 on spreading of SARS-CoV-2 in tropical and temperate zone countries. *Environmental Pollution*, *266*, 115176. https://doi.org/10.1016/j.envpol.2020.115176
11. Dales, R., Blanco-Vidal, C., Romero-Meza, R., Schoen, S., Lukina, A., & Cakmak, S. (2021). The association between air pollution and COVID-19 related mortality in Santiago, Chile: A daily time series analysis. *Environmental Research*, *198*, 111284. https://doi.org/10.1016/j.envres.2021.111284
12. Fernández, D., Giné-Vázquez, I., Liu, I., Yucel, R., Nai Ruscone, M., Morena, M., García, V. G., Haro, J. M., Pan, W., & Tyrovolas, S. (2021). Are environmental pollution and biodiversity levels associated to the spread and mortality of COVID-19? A four-month global analysis. *Environmental Pollution*, *271*, 116326. https://doi.org/10.1016/j.envpol.2020.116326
13. Gautam, S., Talatiya, A., Patel, M., Chabhadiya, K., & Pathak, P. (2020). Personal Exposure to Air Pollutants from Winter Season Bonfires in Rural Areas of Gujarat, India. *Exposure and Health*, *12*(1), 89–97. https://doi.org/10.1007/s12403-018-0287-9
14. Golub, A., & Strukova, E. (2008). Evaluation and Identification of Priority Air Pollutants for Environmental Management on the Basis of Risk Analysis in Russia. *Journal of Toxicology and Environmental Health, Part A*, *71*(1), 86–91. https://doi.org/10.1080/15287390701558238
15. Hoang, T., & Tran, T. T. A. (2021). Ambient air pollution, meteorology, and COVID‐19 infection in Korea. *Journal of Medical Virology*, *93*(2), 878–885. https://doi.org/10.1002/jmv.26325
16. Huh, K., Hong, J., & Jung, J. (2020). Association of meteorological factors and atmospheric particulate matter with the incidence of pneumonia: An ecological study. *Clinical Microbiology and Infection*, *26*(12), 1676–1683. https://doi.org/10.1016/j.cmi.2020.03.006
17. Ion-Nedelcu, N., Niţescu, M., Caian, M., Bacruban, R., & Ceauşu, E. (2008). [Effect of air pollution upon the hospitalization for acute lower respiratory tract infections among the Bucharest municipality’s residents]. *Bacteriologia, Virusologia, Parazitologia, Epidemiologia (Bucharest, Romania: 1990)*, *53*(2), 117–120.
18. Kalmykova, G. N., & Popova, A. I. (1994). [Effect of environmental pollution on children and adolescents with increased risk for tuberculosis infection]. *Problemy Tuberkuleza*, *2*, 12–14.
19. Khauadamova, G. T., & Kumisbaeva, B. T. (1997). [Influence of environmental chemical factors on the risk for different types of pulmonary tuberculosis]. *Problemy Tuberkuleza*, *5*, 19–21.
20. Kumar, M., & Tiwari, R. R. (2019). *Recent Trends and Advances in Environmental Health* (illustrated ed.). Nova Science Publishers. https://books.google.com.bd/books/about/Recent_Trends_and_Advances_in_Environmen.html?id=dvo2xAEACAAJ&redir_esc=y
21. Liu, Q., Xu, S., & Lu, X. (2021). Association between air pollution and COVID-19 infection: Evidence from data at national and municipal levels. *Environmental Science and Pollution Research*, *28*(28), 37231–37243. https://doi.org/10.1007/s11356-021-13319-5
22. Lym, Y., & Kim, K.-J. (2022). Exploring the effects of PM2.5 and temperature on COVID-19 transmission in Seoul, South Korea. *Environmental Research*, *203*, 111810. https://doi.org/10.1016/j.envres.2021.111810
23. Memish, Z. A., Almasri, M., Assirri, A., Al-Shangiti, A. M., Gray, G. C., Lednicky, J. A., & Yezli, S. (2014). Environmental sampling for respiratory pathogens in Jeddah airport during the 2013 Hajj season. *American Journal of Infection Control*, *42*(12), 1266–1269. https://doi.org/10.1016/j.ajic.2014.07.027
24. Meo, S. A., Al-Khlaiwi, T., & Ullah, C. H. (2021). Effect of ambient air pollutants PM2.5 and PM10 on COVID-19 incidence and mortality: Observational study. *European Review for Medical and Pharmacological Sciences*, *25*(23), 7553–7564. https://doi.org/10.26355/eurrev_202112_27455
25. Meo, S. A., Almutairi, F. J., Abukhalaf, A. A., Alessa, O. M., Al-Khlaiwi, T., & Meo, A. S. (2021). Sandstorm and its effect on particulate matter PM 2.5, carbon monoxide, nitrogen dioxide, ozone pollutants and SARS-CoV-2 cases and deaths. *Science of The Total Environment*, *795*, 148764. https://doi.org/10.1016/j.scitotenv.2021.148764
26. Onishchenko, G. G., Mamaev, I. A., & Guseĭnov, G. K. (2006). [Impact of the area burden of agrochemicals on tuberculosis morbidity and mortality]. *Problemy Tuberkuleza I Boleznei Legkikh*, *7*, 30–33.
27. Orak, N. H., & Ozdemir, O. (2021). The impacts of COVID-19 lockdown on PM10 and SO2 concentrations and association with human mobility across Turkey. *Environmental Research*, *197*, 111018. https://doi.org/10.1016/j.envres.2021.111018
28. Rashidi, R., Khaniabadi, Y. O., Sicard, P., De Marco, A., & Anbari, K. (2023). Ambient PM2.5 and O3 pollution and health impacts in Iranian megacity. *Stochastic Environmental Research and Risk Assessment*, *37*(1), 175–184. https://doi.org/10.1007/s00477-022-02286-z
29. Repetto, R., & Baliga, S. S. (1997). Pesticides and Immunosuppression: The Risks to Public Health. *Health Policy and Planning*, *12*(2), 97–106. https://doi.org/10.1093/heapol/12.2.97
30. Reyes, M. S. S., & Medina, P. M. B. (2020). Environmental pollutant exposure can exacerbate COVID-19 neurologic symptoms. *Medical Hypotheses*, *144*, 110136. https://doi.org/10.1016/j.mehy.2020.110136
31. Safo-Adu, G., Attiogbe, F., Emahi, I., & Ofosu, F. G. (2023). Outdoor and indoor particle air pollution and its health consequences in African cities: New evidence and an exhortation. *Sustainable Environment*, *9*(1), 2265729. https://doi.org/10.1080/27658511.2023.2265729
32. Semczuk-Kaczmarek, K., Rys-Czaporowska, A., Sierdzinski, J., Kaczmarek, L. D., Szymanski, F. M., & Platek, A. E. (2022). Association between air pollution and COVID-19 mortality and morbidity. *Internal and Emergency Medicine*, *17*(2), 467–473. https://doi.org/10.1007/s11739-021-02834-5
33. Shamsiyev, A. M., & Khusinova, Sh. A. (2008). The Influence of Environmental Factors on Human Health in Uzbekistan. In R. Behnke (Ed.), *The Socio-Economic Causes and Consequences of Desertification in Central Asia* (pp. 249–252). Springer Netherlands. https://doi.org/10.1007/978-1-4020-8544-4_11
34. Shim, S. R., Kim, H. J., Hong, M., Kwon, S. K., Kim, J. H., Lee, S. J., Lee, S. W., & Han, H. W. (2022). Effects of meteorological factors and air pollutants on the incidence of COVID-19 in South Korea. *Environmental Research*, *212*, 113392. https://doi.org/10.1016/j.envres.2022.113392
35. Sohn, M., Kim, H., Sung, H., Lee, Y., Choi, H., & Chung, H. (2019). Association of social deprivation and outdoor air pollution with pulmonary tuberculosis in spatiotemporal analysis. *International Journal of Environmental Health Research*, *29*(6), 657–667. https://doi.org/10.1080/09603123.2019.1566522
36. Thapa, K., & Pant, B. R. (2014). Pesticides in vegetable and food commodities: Environment and public health concern. *Journal of Nepal Health Research Council*, *12*(28), 208–210.
37. Toczylowski, K., Wietlicka-Piszcz, M., Grabowska, M., & Sulik, A. (2021). Cumulative Effects of Particulate Matter Pollution and Meteorological Variables on the Risk of Influenza-Like Illness. *Viruses*, *13*(4), 556. https://doi.org/10.3390/v13040556
