# Supplementary material for: A systematic review of the pivotal role of environmental toxicant exposure on infectious diseases in low- and middle-income countries
Source: Public Health Pract (Oxf). 2025 Jun 25;10:100631. doi: 10.1016/j.puhip.2025.100631 (PMC12274766; doi:10.1016/j.puhip.2025.100631)
Supplement: Multimedia component 4 [file mmc4.docx]

# **Table S4: Outcomes and outcome measurements**

| **Author, Year** | **WHO Region** | **Outcome** | **Disease Outcome** | **Measurement** |
| --- | --- | --- | --- | --- |
| (Liang et al., 2014) | Western Pacific | Incidence | Influenza | Correlation |
| (Rivas-Santiago et al., 2015) | Americas | Mortality | Tuberculosis | Correlation |
| (Memon et al., 2017) | South-East Asia | Concentration | Tuberculosis | Concentration |
| (Bates et al., 2018) | South-East Asia | Incidence | ALRI | Odds ratio |
| (Emokpae and Mbonu, 2018) | Africa | Concentration | AIDS | Concentration |
| (Zhu et al., 2018) | Western Pacific | [Incidence](about:blank#_msocom_1) | Tuberculosis | Odds ratio |
| (Zhao et al., 2019) | Western Pacific | Incidence | Pneumonia | Concentration |
| (Mokoena et al., 2019) | Western Pacific | Mortality | Respiratory Disease, Pneumonia, Influenza | Concentration |
| (Dastoorpoor et al., 2019) | Eastern Mediterranean | Incidence | Respiratory Disease | Risk Ratio |
| (Aslam et al., 2019) | South-East Asia | Concentration | Hepatitis C | N/A |
| (BS Zhang and MD Zhang, 2019) | Western Pacific | Incidence | Tuberculosis | Correlation |
| (Davila Cordova et al., 2020) | Americas | Incidence | ALRI, Pneumonia | Risk Ratio |
| (Yuan et al., 2020) | Western Pacific | Incidence | COVID-19 | Correlation |
| (Roux et al., 2020) | Americas | [Incidence](about:blank#_msocom_2) | SARD | Correlation |
| (Ruchiraset and Tantrakarnapa, 2022) | Americas | Incidence | Tuberculosis | Correlation |
| (Wu et al., 2021) | Western Pacific | Morbidity | COVID-19 | Correlation |
| (Zhang et al., 2021) | Western Pacific | Incidence | COVID-19 | Correlation |
| (Meng et al., 2021) | Western Pacific | Incidence | Influenza | Risk Ratio |
| (Carrasco-Escobar et al., 2020) | Western Pacific | Mortality | Respiratory Disease, Pneumonia | Risk Ratio |
| (Zhu et al., 2021) | Western pacific | Incidence | COVID-19 | N/A |
| (Zheng et al., 2021) | Western Pacific | Incidence | COVID-19 | Risk Ratio |
| (Lu et al., 2021) | South-East Asia | Incidence | COVID-19 | N/A |
| (Laxmipriya and Narayanan, 2021) | South-East Asia | Incidence | COVID-19 | Correlation |
| (Sangkham et al., 2021) | Americas | Morbidity | COVID-19 | Correlation |
| (Kutralam-Muniasamy et al., 2021) | South - East Asia | Incidence | COVID-19 | N/A |
| (Sahoo, 2021) | South-East Asia | Incidence | COVID-19 | Correlation |
| (Mehmood et al., 2021) | South-East Asia | Incidence | URI, Pneumonia | Risk Ratio |
| (Páez-Osuna et al., 2022) | Americas | Incidence, Mortality | COVID-19 | Correlation |
| (Sherris et al., 2021) | Western Pacific | Incidence, Mortality and Morbidity | COVID-19 | N/A |
| (Samillan et al., 2021) | Americas | Incidence | COVID-19 | Risk Ratio |
| (Nor et al., 2021) | Western Pacific | Incidence | COVID-19 | Risk Ratio |
| (Wannaz et al., 2021) | South-East Asia | Morbidity | Respiratory Disease | Risk Ratio |
| (Ma et al., 2021, p. 21) | Western Pacific | Incidence, Mortality | Tuberculosis | Correlation |
| (Priyankara et al., 2021) | Americas | Incidence, Mortality and Morbidity | COVID-19 | Correlation |
| (Meo et al., 2022) | Western Pacific | Incidence, Morbidity | Pneumonia | Correlation |
| (Liu et al., 2021) | South-East Asia | Incidence | Pneumonia | Risk Ratio |
| (Vasquez-Apestegui et al., 2021) | Americas | Mortality | COVID-19 | Correlation |
| (Sangkham et al., 2023) | South-East Asia | Incidence, Mortality | COVID-19 | Concentration |
| (Wang et al., 2021) | Global | Incidence | COVID-19 | Correlation |
| (Ali et al., 2022) | South-East Asia | Incidence, Mortality | COVID-19 | Correlation |
| (Popovic et al., 2023) | Western Pacific | Incidence | Pneumonia | Risk Ratio |
| (Meo et al., 2022) | Western Pacific | Incidence | ALRI | Excess Risk |
| (Huihui Zhang et al., 2022) | Eastern Mediterranean | Incidence | COVID-19 | Correlation |
| (Xiao et al., 2022) | South-East Asia | Mortality | COVID-19 | Correlation |
| (Khalis et al., 2022) | South-East Asia | Incidence | Influenza, Pneumonia | Concentration |
| (Khan, 2022) | Americas | Mortality | COVID-19 | Correlation |
| (Jainonthee et al., 2022) | South - East Asia | Incidence | COVID-19 | Concentration |
| (Gonçalves et al., 2023) | Western Pacific | Incidence | Tuberculosis | Risk Ratio |
| (Damasceno et al., 2022) | Americas | Incidence, Mortality | COVID-19 | Risk Ratio |
| (Zheng et al., 2023) | Western Pacific | Incidence, Morbidity | ALRI | Excess Risk |
| (Bonilla et al., 2023) | Americas | Mortality | COVID-19 | Correlation |
| (Wang et al., 2023) | Western Pacific | Humidity | Tuberculosis | Concentration |
| (Chen et al., 2023) | Western Pacific | Incidence | Influenza | Risk Ratio |
| (Nie et al., 2023) | Western Pacific | Incidence | Tuberculosis, URTI, pneumonia, COPD. | Correlation |
| (Luo, 2024) | Western Pacific | Incidence | Tuberculosis | Risk Ratio |

References:

1. Ali, A., Farhan, S. B., Zhang, Y., Nasir, J., Farhan, H., Zamir, U. B., & Gao, H. (2022). Changes in temporal pattern and spatial distribution of environmental pollutants in 8 Asian countries owing to COVID-19 pandemic. *Chemosphere*, *308*, 136075. https://doi.org/10.1016/j.chemosphere.2022.136075
2. Aslam, N., Sarfaraz Iqbal, M., Makhdoom Hussain, S., Rizwan, M., Naseer, Q.-U.-A., Afzal, M., Muneer, R., Batool, F., 1 Department of Biochemistry, Government College University Faisalabad (GCUF), Pakistan, 2 Department of Bioinformatics &amp; Computational biology, Virtual University of Pakistan, Pakistan, 3 Department of Zoology, Government College University Faisalabad (GCUF), Pakistan, 4 Department of Statistics, Government College University, Faisalabad, Pakistan, & 5 Health Care Center Government College University Faisalabad (GCUF), Pakistan. (2019). Effects of chelating agents on heavy metals in Hepatitis C Virus (HCV) patients. *Mathematical Biosciences and Engineering*, *16*(3), 1138–1149. https://doi.org/10.3934/mbe.2019054
3. Bates, M. N., Pokhrel, A. K., Chandyo, R. K., Valentiner-Branth, P., Mathisen, M., Basnet, S., Strand, T. A., Burnett, R. T., & Smith, K. R. (2018). Kitchen PM2.5 concentrations and child acute lower respiratory infection in Bhaktapur, Nepal: The importance of fuel type. *Environmental Research*, *161*, 546–553. https://doi.org/10.1016/j.envres.2017.11.056
4. Bonilla, J. A., Lopez-Feldman, A., Pereda, P. C., Rivera, N. M., & Ruiz-Tagle, J. C. (2023). Association between long-term air pollution exposure and COVID-19 mortality in Latin America. *PLOS ONE*, *18*(1), e0280355. https://doi.org/10.1371/journal.pone.0280355
5. BS Zhang, C. Y., & MD Zhang, A. (2019). Climate and air pollution alter incidence of tuberculosis in Beijing, China. *Annals of Epidemiology*, *37*, 71–76. https://doi.org/10.1016/j.annepidem.2019.07.003
6. Carrasco-Escobar, G., Schwalb, A., Tello-Lizarraga, K., Vega-Guerovich, P., & Ugarte-Gil, C. (2020). Spatio-temporal co-occurrence of hotspots of tuberculosis, poverty and air pollution in Lima, Peru. *Infectious Diseases of Poverty*, *9*(1), 32. https://doi.org/10.1186/s40249-020-00647-w
7. Chen, Y., Hou, W., Hou, W., & Dong, J. (2023). Lagging effects and prediction of pollutants and their interaction modifiers on influenza in northeastern China. *BMC Public Health*, *23*(1). https://doi.org/10.1186/s12889-023-16712-6
8. Damasceno, R. M., Cicerelli, R. E., Almeida, T. D., & Requia, W. J. (2022). Air Pollution and COVID-19 Mortality in Brazil. *Atmosphere*, *14*(1), 5. https://doi.org/10.3390/atmos14010005
9. Dastoorpoor, M., Masoumi, K., Vahedian, M., Aghababaeian, H., Sekhavatpour, Z., Khanjani, N., & Idani, E. (2019). Associations of short-term exposure to air pollution with respiratory hospital admissions in Ahvaz, Iran. *Process Safety and Environmental Protection*, *123*, 150–160. https://doi.org/10.1016/j.psep.2019.01.012
10. Davila Cordova, J. E., Tapia Aguirre, V., Vasquez Apestegui, V., Ordoñez Ibarguen, L., Vu, B. N., Steenland, K., & Gonzales, G. F. (2020). Association of PM2.5 concentration with health center outpatient visits for respiratory diseases of children under 5 years old in Lima, Peru. *Environmental Health*, *19*(1), 7. https://doi.org/10.1186/s12940-020-0564-5
11. Emokpae, M. A., & Mbonu, I. (2018). Blood levels of some toxic metals in Human Immunodeficiency Virus (HIV) Type 1- infection. *Annals of Health Research*, *4*(1), 75–81. https://doi.org/10.30442/ahr.0401-1-10
12. Gonçalves, K. D. S., Cirino, G. G., Costa, M. O. D., Couto, L. D. O. D., Tortelote, G. G., & Hacon, S. D. S. (2023). The potential impact of PM2.5 on the covid-19 crisis in the Brazilian Amazon region. *Revista de Saúde Pública*, *57*(1), 67. https://doi.org/10.11606/s1518-8787.2023057005134
13. Huihui Zhang, Liu, S., Dou, Q., Huang, Z., Lv, C., Liao, J., Tao, F., Liu, Y., & Ma, L. (2022). Association between Ambient Air Pollutants and Pneumonia in Wuhan, China, 2014–2017. *Atmosphere*, *13*(4), 578. https://doi.org/10.3390/atmos13040578
14. Jainonthee, C., Wang, Y.-L., Chen, C. W. K., & Jainontee, K. (2022). Air Pollution-Related Respiratory Diseases and Associated Environmental Factors in Chiang Mai, Thailand, in 2011–2020. *Tropical Medicine and Infectious Disease*, *7*(11), 341. https://doi.org/10.3390/tropicalmed7110341
15. Khalis, M., Toure, A. B., El Badisy, I., Khomsi, K., Najmi, H., Bouaddi, O., Marfak, A., Al-Delaimy, W. K., Berraho, M., & Nejjari, C. (2022). Relationship between Meteorological and Air Quality Parameters and COVID-19 in Casablanca Region, Morocco. *International Journal of Environmental Research and Public Health*, *19*(9), 4989. https://doi.org/10.3390/ijerph19094989
16. Khan, Y. A. (2022). Risk of mortality due to COVID-19 and air pollution in Pakistan. *Environmental Science and Pollution Research*, *29*(2), 2063–2072. https://doi.org/10.1007/s11356-021-15654-z
17. Kutralam-Muniasamy, G., Pérez-Guevara, F., Martínez, I. E., & Chari, S. V. (2021). Particulate matter concentrations and their association with COVID-19-related mortality in Mexico during June 2020 Saharan dust event. *Environmental Science and Pollution Research*, *28*(36), 49989–50000. https://doi.org/10.1007/s11356-021-14168-y
18. Laxmipriya, S., & Narayanan, Rm. (2021). COVID-19 and its relationship to particulate matter pollution – Case study from part of greater Chennai, India. *Materials Today: Proceedings*, *43*, 1634–1639. https://doi.org/10.1016/j.matpr.2020.09.768
19. Liang, Y., Fang, L., Pan, H., Zhang, K., Kan, H., Brook, J. R., & Sun, Q. (2014). PM2.5 in Beijing – temporal pattern and its association with influenza. *Environmental Health*, *13*(1), 102. https://doi.org/10.1186/1476-069X-13-102
20. Liu, Q., Xu, S., & Lu, X. (2021). Association between air pollution and COVID-19 infection: Evidence from data at national and municipal levels. *Environmental Science and Pollution Research*, *28*(28), 37231–37243. https://doi.org/10.1007/s11356-021-13319-5
21. Lu, B., Wu, N., Jiang, J., & Li, X. (2021). Associations of acute exposure to airborne pollutants with COVID-19 infection: Evidence from China. *Environmental Science and Pollution Research*, *28*(36), 50554–50564. https://doi.org/10.1007/s11356-021-14159-z
22. Luo, D., Wang, L., Zhang, M., Martinez, L., Chen, S., Zhang, Y., Wang, W., Wu, Q., Wu, Y., Liu, K., Xie, B., & Chen, B. (2024). Spatial spillover effect of environmental factors on the tuberculosis occurrence among the elderly: A surveillance analysis for nearly a dozen years in eastern China. *BMC Public Health*, *24*(1). https://doi.org/10.1186/s12889-024-17644-5
23. Ma, Y., Cheng, B., Shen, J., Wang, H., Feng, F., Zhang, Y., & Jiao, H. (2021). Association between environmental factors and COVID-19 in Shanghai, China. *Environmental Science and Pollution Research*, *28*(33), 45087–45095. https://doi.org/10.1007/s11356-021-13834-5
24. Mehmood, K., Bao, Y., Abrar, M. M., Petropoulos, G. P., Saifullah, Soban, A., Saud, S., Khan, Z. A., Khan, S. M., & Fahad, S. (2021). Spatiotemporal variability of COVID-19 pandemic in relation to air pollution, climate and socioeconomic factors in Pakistan. *Chemosphere*, *271*, 129584. https://doi.org/10.1016/j.chemosphere.2021.129584
25. Memon, Z. M., Yilmaz, E., Shah, A. M., Sahin, U., Kazi, T. G., Devrajani, B. R., & Soylak, M. (2017). Trace elements in blood samples of smoker and nonsmoker active pulmonary tuberculosis patients from Jamshoro, Pakistan. *Environmental Science and Pollution Research*, *24*(34), 26513–26520. https://doi.org/10.1007/s11356-017-0236-3
26. Meng, Y., Lu, Y., Xiang, H., & Liu, S. (2021). Short-term effects of ambient air pollution on the incidence of influenza in Wuhan, China: A time-series analysis. *Environmental Research*, *192*, 110327. https://doi.org/10.1016/j.envres.2020.110327
27. Meo, S. A., Ahmed Alqahtani, S., Saad Binmeather, F., Abdulrhman AlRasheed, R., Mohammed Aljedaie, G., & Mohammed Albarrak, R. (2022). Effect of environmental pollutants PM2.5, CO, O3 and NO2, on the incidence and mortality of SARS-COV-2 in largest metropolitan cities, Delhi, Mumbai and Kolkata, India. *Journal of King Saud University - Science*, *34*(1), 101687. https://doi.org/10.1016/j.jksus.2021.101687
28. Meo, S. A., Shafi, K. M., Al-Masri, A. A., Al-Khlaiwi, T., Alshahrani, A. N., Ejaz, S., Alrassan, L. A., & Yaqinuddin, A. (2022). Public health in Global South: Effect of environmental pollutant PM2.5 on the incidence and mortality of SARS-CoV-2 in Karachi, Lahore, and Islamabad. *European Review for Medical and Pharmacological Sciences*, *26*(23), 9054–9060. https://doi.org/10.26355/eurrev_202212_30581
29. Mokoena, K. K., Ethan, C. J., Yu, Y., Shale, K., & Liu, F. (2019). Ambient air pollution and respiratory mortality in Xi’an, China: A time-series analysis. *Respiratory Research*, *20*(1), 139. https://doi.org/10.1186/s12931-019-1117-8
30. Nie, T., Chen, J., Ji, Y., Lin, T., & Wang, J. (2023). Impact of Air Pollution on Respiratory Diseases in Typical Industrial City in the North China Plain. *Sustainability*, *15*(14), 11198. https://doi.org/10.3390/su151411198
31. Nor, N. S. M., Yip, C. W., Ibrahim, N., Jaafar, M. H., Rashid, Z. Z., Mustafa, N., Hamid, H. H. A., Chandru, K., Latif, M. T., Saw, P. E., Lin, C. Y., Alhasa, K. M., Hashim, J. H., & Nadzir, M. S. M. (2021). Particulate matter (PM2.5) as a potential SARS-CoV-2 carrier. *Scientific Reports*, *11*(1). https://doi.org/10.1038/s41598-021-81935-9
32. Páez-Osuna, F., Valencia-Castañeda, G., & Rebolledo, U. A. (2022). The link between COVID-19 mortality and PM2.5 emissions in rural and medium-size municipalities considering population density, dust events, and wind speed. *Chemosphere*, *286*, 131634. https://doi.org/10.1016/j.chemosphere.2021.131634
33. Popovic, I., Soares Magalhães, R. J., Yang, Y., Yang, S., Yang, B., Dong, G., Wei, X., Fox, G. J., Hammer, M. S., Martin, R. V., Van Donkelaar, A., Ge, E., Marks, G. B., & Knibbs, L. D. (2023). Effects of long-term ambient air pollution exposure on township-level pulmonary tuberculosis notification rates during 2005–2017 in Ningxia, China. *Environmental Pollution*, *317*, 120718. https://doi.org/10.1016/j.envpol.2022.120718
34. Priyankara, S., Senarathna, M., Jayaratne, R., Morawska, L., Abeysundara, S., Weerasooriya, R., Knibbs, L. D., Dharmage, S. C., Yasaratne, D., & Bowatte, G. (2021). Ambient PM2.5 and PM10 Exposure and Respiratory Disease Hospitalization in Kandy, Sri Lanka. *International Journal of Environmental Research and Public Health*, *18*(18), 9617. https://doi.org/10.3390/ijerph18189617
35. Rivas-Santiago, C. E., Sarkar, S., Cantarella, P., Osornio-Vargas, Á., Quintana-Belmares, R., Meng, Q., Kirn, T. J., Ohman Strickland, P., Chow, J. C., Watson, J. G., Torres, M., & Schwander, S. (2015). Air Pollution Particulate Matter Alters Antimycobacterial Respiratory Epithelium Innate Immunity. *Infection and Immunity*, *83*(6), 2507–2517. https://doi.org/10.1128/IAI.03018-14
36. Roux, E., Ignotti, E., Bègue, N., Bencherif, H., Catry, T., Dessay, N., Gracie, R., Gurgel, H., de Sousa Hacon, S., de A. F. M. Magalhães, M., Vieira Monteiro, A. M., Revillion, C., Antunes Maciel Villela, D., Xavier, D., & Barcellos, C. (2020). Toward an Early Warning System for Health Issues Related to Particulate Matter Exposure in Brazil: The Feasibility of Using Global PM2.5 Concentration Forecast Products. *Remote Sensing*, *12*(24), 4074. https://doi.org/10.3390/rs12244074
37. Ruchiraset, A., & Tantrakarnapa, K. (2022). Association of climate factors and air pollutants with pneumonia incidence in Lampang province, Thailand: Findings from a 12-year longitudinal study. *International Journal of Environmental Health Research*, *32*(3), 691–700. https://doi.org/10.1080/09603123.2020.1793919
38. Sahoo, M. M. (2021). Significance between air pollutants, meteorological factors, and COVID-19 infections: Probable evidences in India. *Environmental Science and Pollution Research*, *28*(30), 40474–40495. https://doi.org/10.1007/s11356-021-12709-z
39. Samillan, V. J., Flores-León, D., Rojas, E., & Zutta, B. R. (2021). Environmental and climatic impact on the infection and mortality of SARS-CoV-2 in Peru. *Journal of Basic and Clinical Physiology and Pharmacology*, *32*(5), 935–942. https://doi.org/10.1515/jbcpp-2021-0007
40. Sangkham, S., Islam, Md. A., Sarndhong, K., Vongruang, P., Hasan, M. N., Tiwari, A., & Bhattacharya, P. (2023). Effects of fine particulate matter (PM2.5) and meteorological factors on the daily confirmed cases of COVID-19 in Bangkok during 2020–2021, Thailand. *Case Studies in Chemical and Environmental Engineering*, *8*, 100410. https://doi.org/10.1016/j.cscee.2023.100410
41. Sangkham, S., Thongtip, S., & Vongruang, P. (2021). Influence of air pollution and meteorological factors on the spread of COVID-19 in the Bangkok Metropolitan Region and air quality during the outbreak. *Environmental Research*, *197*, 111104. https://doi.org/10.1016/j.envres.2021.111104
42. Sherris, A. R., Begum, B. A., Baiocchi, M., Goswami, D., Hopke, P. K., Brooks, W. A., & Luby, S. P. (2021). Associations between ambient fine particulate matter and child respiratory infection: The role of particulate matter source composition in Dhaka, Bangladesh. *Environmental Pollution*, *290*, 118073. https://doi.org/10.1016/j.envpol.2021.118073
43. Vasquez-Apestegui, B. V., Parras-Garrido, E., Tapia, V., Paz-Aparicio, V. M., Rojas, J. P., Sanchez-Ccoyllo, O. R., & Gonzales, G. F. (2021). Association between air pollution in Lima and the high incidence of COVID-19: Findings from a post hoc analysis. *BMC Public Health*, *21*(1). https://doi.org/10.1186/s12889-021-11232-7
44. Wang, Q., Li, Y., Yin, Y., Hu, B., Yu, C., Wang, Z., Li, Y., Xu, C., & Wang, Y. (2023). Association of air pollutants and meteorological factors with tuberculosis: A national multicenter ecological study in China. *International Journal of Biometeorology*, *67*(10), 1629–1641. https://doi.org/10.1007/s00484-023-02524-1
45. Wang, X., Xu, Z., Su, H., Ho, H. C., Song, Y., Zheng, H., Hossain, M. Z., Khan, M. A., Bogale, D., Zhang, H., Wei, J., & Cheng, J. (2021). Ambient particulate matter (PM1, PM2.5, PM10) and childhood pneumonia: The smaller particle, the greater short-term impact? *Science of The Total Environment*, *772*, 145509. https://doi.org/10.1016/j.scitotenv.2021.145509
46. Wannaz, E. D., Larrea Valdivia, A. E., Reyes Larico, J. A., Salcedo Peña, J., & Valenzuela Huillca, C. (2021). PM10 correlates with COVID-19 infections 15 days later in Arequipa, Peru. *Environmental Science and Pollution Research*, *28*(29), 39648–39654. https://doi.org/10.1007/s11356-021-13408-5
47. Wu, Y., Zhan, Q., & Zhao, Q. (2021). Long-term Air Pollution Exposure Impact on COVID-19 Morbidity in China. *Aerosol and Air Quality Research*, *21*(1), 200413. https://doi.org/10.4209/aaqr.2020.07.0413
48. Xiao, D., Guo, W., Xu, D., Chen, J., Liang, Z., & Zhang, X. (2022). Three Exposure Metrics for Fine Particulate Matter Associated With Outpatient Visits for Acute Lower Respiratory Infection Among Children in Guangzhou, China. *Frontiers in Public Health*, *10*. https://doi.org/10.3389/fpubh.2022.876496
49. Yuan, W., Fulgar, C. C., Sun, X., Vogel, C. F. A., Wu, C.-W., Zhang, Q., Bein, K. J., Young, D. E., Li, W., Wei, H., & Pinkerton, K. E. (2020). In vivo and in vitro inflammatory responses to fine particulate matter (PM2.5) from China and California. *Toxicology Letters*, *328*, 52–60. https://doi.org/10.1016/j.toxlet.2020.04.010
50. Zhang, X., Tang, M., Guo, F., Wei, F., Yu, Z., Gao, K., Jin, M., Wang, J., & Chen, K. (2021). Associations between air pollution and COVID-19 epidemic during quarantine period in China. *Environmental Pollution*, *268*, 115897. https://doi.org/10.1016/j.envpol.2020.115897
51. Zhao, Y., Hu, J., Tan, Z., Liu, T., Zeng, W., Li, X., Huang, C., Wang, S., Huang, Z., & Ma, W. (2019). Ambient carbon monoxide and increased risk of daily hospital outpatient visits for respiratory diseases in Dongguan, China. *The Science of the Total Environment*, *668*, 254–260. https://doi.org/10.1016/j.scitotenv.2019.02.333
52. Zheng, P., Chen, Z., Liu, Y., Song, H., Wu, C.-H., Li, B., Kraemer, M. U. G., Tian, H., Yan, X., Zheng, Y., Stenseth, N. Chr., & Jia, G. (2021). Association between coronavirus disease 2019 (COVID-19) and long-term exposure to air pollution: Evidence from the first epidemic wave in China. *Environmental Pollution*, *276*, 116682. https://doi.org/10.1016/j.envpol.2021.116682
53. Zheng, Y., Chen, S., Chen, Y., Li, J., Xu, B., Shi, T., & Yang, Q. (2023). Association between PM2.5-bound metals and pediatric respiratory health in Guangzhou: An ecological study investigating source, health risk, and effect. *Frontiers in Public Health*, *11*. https://doi.org/10.3389/fpubh.2023.1137933
54. Zhu, F., Chen, L., Qian, Z., Liao, Y., Zhang, Z., McMillin, S. E., Wang, X., & Lin, H. (2021). Acute effects of particulate matter with different sizes on respiratory mortality in Shenzhen, China. *Environmental Science and Pollution Research*, *28*(28), 37195–37203. https://doi.org/10.1007/s11356-021-13118-y
55. Zhu, S., Xia, L., Wu, J., Chen, S., Chen, F., Zeng, F., Chen, X., Chen, C., Xia, Y., Zhao, X., & Zhang, J. (2018). Ambient air pollutants are associated with newly diagnosed tuberculosis: A time-series study in Chengdu, China. *Science of The Total Environment*, *631–632*, 47–55. https://doi.org/10.1016/j.scitotenv.2018.03.017
