# Supplementary material for: A systematic review of the pivotal role of environmental toxicant exposure on infectious diseases in low- and middle-income countries
Source: Public Health Pract (Oxf). 2025 Jun 25;10:100631. doi: 10.1016/j.puhip.2025.100631 (PMC12274766; doi:10.1016/j.puhip.2025.100631)
Supplement: Multimedia component 6 [file mmc6.docx]

**Table_S6: Toxicants addressed in the associated studies**

| **Toxicants** | **Associated studies** |
| --- | --- |
| **PM 2.5** | Liang 2014, Rivas-Santiago 2015, Bates 2018, Mokoena 2019, Dastoorpoor 2019, Davila Cordova 2020, Carrasco-Escobar 2020, Yuan 2020, Roux 2020, Wu 2021, Laxmipriya 2021,  Zhang 2021, Zhu 2021, Meng 2021, Lu 2021, Shangkham 2021,  Zheng 2021, Kutralam-Muniasamy 2021, Mehmood 2021, Sherris 2021, Sahoo 2021, Samillan 2021, Ma 2021, Liu 2021, Priyankara 2021, Nor 2021, Vasquez-Apestegui 2021, Wannaz 2021, Wang 2021, Khan 2022, Meo 2022, Huihui Zhang 2022, Damasceno 2022, Páez-Osuna 2022, Xiao 2022, Jainonthee 2022, Popovic 2023, Sangkham 2023, Gonçalves 2023, Zheng 2023, Wang 2023, Chen 2023, Nie 2023, Luo 2024 |
| **PM1** | Zhu 2021, Wang 2021 |
| **PM10** | Zhu 2018, Zhang 2019, Yuan 2020, Wu 2021, Zhang 2021, Meng 2021, Zheng 2021, Laxmipriya 2021, Shangkham 2021, Wannaz 2021, Ma 2021, Kutralam-Muniasamy 2021, Wang 2021, Sahoo 2021, Priyankara 2021, Ruchiraset 2022, Khan 2022, Khalis 2022, Jainonthee 2022, Wang 2023, Chen 2023, Nie 2023 |
| **NO2** | Zhu 2018, Zhang 2019, Yuan 2020, Wu 2021, Zhang 2021, Ma 2021, Meng 2021, Lu 2021, Zheng 2021, Sahoo 2021, Samillan 2021, Liu 2021, Meo 2022, Ruchiraset 2022, Khan 2022, Ali 2022, Huihui Zhang 2022, Khalis 2022, Damasceno 2022, Popovic 2023, Wang 2023, Chen 2023, Nie 2023 |
| **O3** | Mokoena 2019, Wu 2021, Zhang 2021, Meng 2021, Lu 2021, Ma 2021, Liu 2021, Ruchiraset 2022, Meo 2022, Damasceno 2022, Khalis 2022, Popovic 2023, Wang 2023 |
| **SO2** | Zhu 2018, Mokoena 2019, Zhang 2019, Yuan 2020, Wu 2021, Zhang 2021, Lu 2021, Sahoo 2021, Meng 2021, Liu 2021, Ma 2021, Ruchiraset 2022, Huihui Zhang 2022, Khalis 2022, Wang 2023, Nie 2023, Luo 2024 |
| **CO** | Zhao 2019, Yuan 2020, Wu 2021, Zhang 2021, Meng 2021, Ma 2021, Liu 2021, Meo 2022, Ruchiraset 2022, Khalis 2022, Wang 2023 |
| **Others** | Emokpae 2018, Aslam 2019, Zheng 2023, Bonilla 2023 |
